# Multi-omics analyses to assess the effects of multi-layer cultivation with supplemental LED lighting on potato yield and quality

**DOI:** 10.3389/fpls.2026.1913161

**Published:** 2026-08-19

**Authors:** Antonio Pannico, Luigi Giuseppe Duri, Sabrina De Pascale, Nafiou Arouna, Antonio Dario Troise, Rosalia Ferracane, Mariapia Esposito, Simonetta Caira, Antonio Giandonato Caporale, Luca Cimmino, Riccardo Aversano, Andrea Scaloni, Stefania De Pascale, Roberta Paradiso

**Affiliations:** 1 Department of Agricultural Sciences, University of Naples Federico II, Naples, Italy; 2 Department of Agriculture, Food, Natural Resources and Engineering (DAFNE), University of Foggia, Foggia, Italy; 3 Institute for the Animal Production System in the Mediterranean Environment (ISPAAM), National Research Council (CNR), Portici, Italy; 4 Institute of Agricultural Biology and Biotechnology, National Research Council (CNR), Pisa, Italy

**Keywords:** bioregenerative life support systems, glycoalkaloids, light spectrum, metabolomics, *Solanum tuberosum* L., tuber quality, vertical greenhouse cultivation

## Abstract

Integrating a vertical layout into greenhouse allows space-efficient cultivation and energy saving; however, shading of the lower canopy makes supplemental artificial lighting necessary. Here, we investigated the effect of red–blue LED supplementation in a vertical greenhouse system for potato cultivation on plant growth, tuber yield, and mineral and metabolite composition, compared to full natural sunlight as a control. Cultivation units were designed to simulate a multiple-layer growing system, in which the uppermost level received only natural light, while LEDs integrated reduced light intensity in the shaded lower level up to 300 µmol m^-2^ s^-1^. Despite the lower daily light integral, LED-supplemented plants showed higher photosynthetic activity and slower decline in leaf chlorophyll content over time, which contributed to higher tuber yield compared to the control. Multi-omics analyses on tuber tissues revealed coordinated metabolic reprogramming. Transcriptomic data showed up-regulation of genes involved in photosynthesis, sugar metabolism, and polyamine biosynthesis in LED-treated plants, together with down-regulation of stress-related genes. Proteomic and metabolomic profiling partially confirmed such trends, highlighting a down-representation of stress-responsive proteins as well as reduced levels of polyphenols, glycoalkaloids and enzymes related to their biosynthesis, highlighting an overall metabolic shift toward an attenuated stress response. In parallel, plants grown under LED lighting showed augmented content of nitrogen, polyamines and some precursor amino acids in the corresponding tubers, qualifying their nutritional characteristics. These results provide mechanistic insights into the relationship between potato plants artificial lighting and molecular reprogramming with the aim of improving crop production and food quality.

## 1 Introduction

Vertical farming (VF) allows space-efficient cultivation by stacking multiple crop layers (van Delden et al., 2021). In a fully controlled environment, VF offers year-round production, high yield, and low risk of plant stress, but it implies high energy costs, particularly for lighting (Van Gerrewey et al., 2021). In greenhouse horticulture, a significant increase in productivity and relevant energy savings could be achieved through vertical racks designed to optimize the use of cultivated area and natural light. However, shading lower canopy layers limits the light availability, making artificial supplemental lighting necessary to maintain sufficient daily light integral (DLI), a key determinant of photosynthetic assimilation, hence of plant growth and yield. DLI also modulates carbon assimilation and downstream primary and secondary metabolism, with the biosynthesis of compounds associated with product quality (van Delden et al., 2021). Implementing supplemental lighting to compensate for the reduced natural radiation should ensure fulfillment of specific light requirements for the optimal growth and development of different crops (Poorter et al., 2019).

Light intensity, spectrum, and photoperiod significantly influence crop productivity as well as the nutritional and functional value of plant-based food (Devlin et al., 2007). Regarding light spectrum, red (R) and blue (B) wavelengths efficiently sustain photosynthesis and biomass accumulation, promote stem elongation and leaf expansion, and can influence seed germination, the process of dormancy and plant flowering. The B light regulates stomatal opening, chlorophyll and secondary metabolite biosynthesis and minimizes shade avoidance responses like excessive stem elongation (Mitchell and Stutte, 2015). The combination of R and B lights has been reported to be beneficial for several crops to optimize plant growth and energy use (Bian et al., 2015).

Light-Emitting Diodes (LEDs) rapidly gained popularity in horticulture due to their significant potential for energy saving, high efficiency in terms of electrical conversion and numerous advantages over traditional lamps, including the possibility to fine-tune light intensity and spectrum to suit specific crop needs in the various phenological phases (Cocetta et al., 2017). LEDs are ideal for VF applications due to their small volume and low heat emission, spectral precision, energy efficiency (Bantis et al., 2018).

VF is ideal for cultivating species with short growth cycle, compact size, and adaptation to high planting density and low light intensity (Kozai and Niu, 2020). These species often show high nutritional and nutraceutical value. Leafy vegetables are the most cultivated crops in VF, with lettuce as the predominant species, followed by cabbage and basil. Conversely, staple crops are less common since they require large cultivation volumes and have a longer growth cycle, hence only a few data are available for their performance in VF.

Potato (*Solanum tuberosum* L.) is one of the most common staple crops in the world after maize, wheat, and rice (https://www.fao.org/faostat); tubers are important source of carbohydrates, proteins, dietary fiber, minerals, vitamins and bioactive compounds, and have a low-fat content (Raigond et al., 2020). Potato is also a candidate crop for space cultivation, thanks to agronomic and nutritional aspects, among which the availability of a wide variety of genotypes, the high harvest index (as edible biomass/total biomass ratio) and the ease of tuber processing (Wheeler, 2006). In potato, it is known that plant growth, yield and tuber quality are influenced by numerous factors including genotype, and environmental and growing conditions. In view of potato integration in Bioregenerative Life Support Systems (BLSSs) for space explorations, essential for sustaining human life in long-term missions, several tests on plant response to different environmental and cultural conditions have been carried out in controlled environments by different space agencies, including National Aeronautics and Space Administration (NASA) and European Space Agency (ESA). Results showed a good adaptation of potato plants to hydroponic cultivation in controlled environment (Wheeler, 2006; Molders et al., 2012), under different artificial light sources (Paradiso et al., 2019), and in pot in different substrates (Paradiso et al., 2019; Caporale et al., 2023). Vertical layout would also mimic the limited lighting conditions expected in future space greenhouses (Nguyen et al., 2023). However, potato has been barely explored for VF cultivation due to plant space and time requirement; further, no data are available on the use of LED-integrated lighting of potato plants in vertical greenhouse systems.

This study has investigated the physiological, metabolic, and molecular responses of potato to greenhouse cultivation under full sunlight and RB LED light supplementation. Plants were placed in two growing units: control plants, simulating the uppermost shelf of cultivation towers, were exposed to full sunlight, while plants representing the lower crop layers, shaded by the overlying shelves, received supplemental RB LED light to maintain a minimum Photosynthetic Photon Flux Density (PPFD) of 300 µmol m^-^² s^-^¹. We evaluated the effects of light quantity and quality on tuber yield as well as mineral and metabolite composition. Multi-omics approaches that combined transcriptomics with proteomics and metabolomics provided an in-depth analysis of the multidimensional link between the genome and the phenotype of potato plants grown under these lighting conditions. This multi-omics approach also deciphered quantitative changes in crucial targets of plant stress response. Incorporation of transcriptomic, proteomic and metabolomic data revealed key genes and their underlying metabolic pathways, clarifying the effect of supplemental LED lighting on plant growth and yield as well as tuber quality in potato grown in a vertical system in greenhouse.

## 2 Materials and methods

### 2.1 Plant material, growth conditions, and experimental design

The experiment was conducted at the Department of Agriculture of the University of Naples Federico II (Portici, Italy; 40° 49’ N, 14°15’ E, 72 m a.s.l.) in a cold greenhouse, from December 20, 2023 to March 7, 2024. Potato (*Solanum tuberosum* L.) cv. ‘Colomba’ (HZPC Holland B.V.) tuber-seeds were pre-sprouted for 15 days in the dark at 18 °C, then transplanted into 5 L transparent plastic pots (20 cm diameter), externally shaded with a black-and-white plastic film to prevent tuber greening. Transparency allowed root inspection during growth. Pots were filled with Agrilit 3 perlite (Perlite Italiana Srl, Corsico, Milan, Italy) and covered with a thin layer of vermiculite to prevent algal formation and substrate dehydration. A complete nutrient solution (pH 5.5, EC 1.8 dS m^-1^) based on the formula proposed by Molders et al. (2012) for potato was used for daily fertigation, returning the evaporate to maintain the substrate moisture close to pot capacity.

Plant density was 13 plants m^-2^ with 25 cm between rows and 30 cm within rows. Two cultivation levels simulated a vertical system: the upper level received only natural light (control, CTRL), while the lower level, shaded by the top shelf, was equipped with a LED panel to supplement the natural light. Temperature and humidity were recorded at the time interval of 30 min using Testo 174H data loggers (Testo Spa, Settimo Milanese, Milan, Italy) positioned at canopy height in each plot.

### 2.2 Lighting treatments

A MEG Phyto 4 LED system (MEG Science, Milan, Italy) with four-channel dimmable LED bars (far red 730 nm, red 660 nm, blue 450 nm and white 5000 K) and a dynamic light control unit was used to set up a red:blue ratio of 2:1. LEDs were dynamically dimmed to integrate the irradiance gap with respect to the CTRL treatment to ensure a minimum PPFD value of 300 µmol m^−2^ s^−1^, to keep the total light intensity in the shaded shelf within the range of 300-400 µmol m^−2^ s^−1^, which was already identified as the best interval for potato cultivation in a controlled environment (Pannico et al., 2022). The dynamic light control unit operated in real time according to the values measured by light sensors placed at the canopy level in both light treatments: only natural light (CTRL) and LED-supplemented natural light (LED). DLI was calculated for each phenological phase (**Supplementary Table 1**).

### 2.3 Net photosynthesis and chlorophyll fluorescence

Measurements were taken at 50, 62, and 76 days after transplanting (DAT), on fully expanded leaves from six plants per treatment. Net photosynthesis (NP) was measured using LCi T compact system (ADC Bioscientific Ltd., Hoddesdon, UK) with RGB LED light at sub-saturating (500 µmol m^−2^ s^−1^) and saturating (1000 µmol m^−2^ s^−1^) light intensities. Ambient CO_2_ was approximately 430 ppm; temperature and humidity averaged 21 °C and 40-45% RH.

Chlorophyll fluorescence (Fv/Fm) was measured with an Opti-Sciences fluorimeter (Plant Stress Kit, Hudson, NY, USA). Saturation pulses were applied at 4286 µmol m^−2^ s^−1^ (Fm’) for light-adapted fluorescence and 3429 µmol m^−2^ s^−1^ (Fm, F0), after 30 min of dark adaptation.

### 2.4 Leaf greenness and stomatal traits

Leaf greenness was assessed using a SPAD-502 chlorophyll meter (Minolta Corp. Ltd., Osaka, Japan). At 62 DAT, abaxial leaf impressions were made on the fifth leaf (from the apex) of six plants per treatment using cyanoacrylate glue and microscope slides. Samples were examined under a VisiScope BL224PLT1 microscope (VWR, Leuven, Belgium). Stomatal density was assessed at 10x in four 1 mm² fields, per observation, with counts averaged for a representative density value. Stomatal size, defined as the length and size (guard cells length/width) was measured at 40x in five stomata per field using ImageJ software (version 1.52a, Wayne Rasband National Institute of Health, Bethesda, MD, USA).

### 2.5 Tuber harvest and samples preparation

At 78 DAT, leaf, stem and tuber number and fresh weight per plant were determined from 12 plants per treatment. Roots were washed, dried at 70 °C until constant weight, and weighed for dry matter.

Leaf area was calculated from Nikon D80 (Nikon Corporation, Tokyo, Japan) digital images using Adobe Photoshop (version 2022, Adobe Incorporated, San Jose, CA, USA) and ImageJ software.

Tubers samples from four plants per treatment were frozen in liquid nitrogen, stored at -80 °C, and lyophilized for phytochemical analysis.

### 2.6 Tuber mineral profile

Freeze-dried tuber samples were ground (IKA, MF10.1, mill, Staufen, Germany). Total N and S were determined from 2 mg samples using an UNICUBE^®^ elemental analyzer (Elementar, Hesse, Germany). For ICP-MS analysis, 100 mg was digested with 2.5 ml HNO_3_ (65%) and 0.5 ml HCl (37%) using a microwave digestion system (Milestone ultra WAVE, Sorisole, BG, Italy). K, P, Mg, Ca, Na, Fe, Mn, Zn, B, Cu and Mo were measured via ICP-MS (iCAP Q, Thermo Scientific, Waltham, MA, USA).

### 2.7 Transcriptomic analysis

Total RNA was extracted from *S. tuberosum* tubers (n = 6, three biological replicates per treatment) using the Spectrum™ Plant Total RNA Kit (Sigma-Aldrich) and assessed for quantity and purity with a NanoDrop™ 2000 spectrophotometer (Thermo Fisher Scientific). Integrity was confirmed by agarose gel electrophoresis. RNA-seq libraries were prepared from high-quality samples and sequenced on an Illumina NovaSeq™ 6000 platform (2 × 150 bp). Raw reads were trimmed using fastp (Chen et al., 2018) and mapped to the *S. tuberosum* reference genome (Stuberosum 686 v6.1) with STAR (Dobin et al., 2013), achieving mapping rates between 86.36% and 88.19%. Strand specificity was assessed with RSeQC (Wang et al., 2012), and gene-level quantification was performed using feature Counts (Liao et al., 2014). Low-abundance transcripts were filtered using HTSFilter (Rau et al., 2013), retaining 22,485 of 32,917 detected genes (68.3%). Differential expression between LED and control treatments was determined using edgeR with an FDR threshold of 0.05. GO and KEGG enrichment analyses were conducted using cluster Profiler, retaining categories with adjusted *p* < 0.05 and a minimum of three differentially expressed genes. Full methodological details, including quality control metrics, filtering parameters, and annotation references, are provided in Supplementary Information (SI). RNA-seq data have been deposited in the NCBI Sequence Read Archive (see Data and code availability).

### 2.8 Protein extraction, identification, and quantitation by mass spectrometry

Proteins were extracted from lyophilized peeled tubers (n = 8, four biological replicates per treatment) using the phenol-based method (Li et al., 2015), with minor modifications. After extraction, samples were proteolyzed with trypsin and resulting digests were analyzed by nano-UHPLC-ESI-Q-Orbitrap-MS/MS using a Vanquish Neo nano-chromatographic system coupled to an Exploris 480 mass spectrometer (Thermo Fisher Scientific, Bremen, Germany). Peptides were separated on an EASY-Spray C18 column (Thermo Fisher Scientific) and raw data were processed using MaxQuant v1.6.8.0 with the integrated Andromeda search engine against the *S. tuberosum* UniProtKB database (Tyanova et al., 2015) applying a false discovery rate (FDR) of 0.01%. Protein quantification data were analyzed with Perseus v1.6.8.0 (FDR < 0.05) (Tyanova et al., 2016). See the Supplementary Information (SI) for details.

Protein–protein interaction (PPI) networks were constructed using the STRING database (https://string-db.org) with a minimum interaction score of 0.4 to ensure medium confidence. Functional enrichment analysis, including Gene Ontology (GO) classification, covering biological processes, molecular functions, and cellular components, was also performed within STRING. Pathway analysis was conducted using the KEGG database via the BlastKOALA tool (https://www.kegg.jp/blastkoala/). The mass spectrometry proteomics data have been deposited to the ProteomeXchange Consortium (see Data and code availability).

### 2.9 Untargeted metabolomic analysis

Freeze-dried tubers (with skin) were ground into powder. Metabolites were extracted using 100 mg sample in 3 mL methanol/water/formic acid (70:29.5:0.5 v/v) according to the procedure described by Troise et al. (2025). After sonication (15 min), vortexing (5 min), and centrifugation (18000xg, 5 min, 4 °C), supernatants were diluted 5-fold and analyzed. Analysis was performed via LC-MS/MS using an Exploris 120 Orbitrap (Thermo Fisher Scientific, Bremen, Germany) linked to a Vanquish Core LC system. A Kinetex biphenyl column (100 x 2.1 mm, 2.6 µm, Phenomenex, Torrance, CA) operated at 35 °C and 0.3 mL/min flow rate. Samples were acquired in polarity switching mode (*m/z* 120-1500), followed by ddMS2 scans with defined collision energies and dynamic exclusion (see SI for experimental details). Raw data were processed in Compound Discoverer 3.3 (Thermo Scientific) for peak detection, alignment, background removal, and identification. Fragmentation spectra were matched against mzCloud, ChemSpider, Phenol-Explorer, Natural Compounds Atlas, and KEGG database. Discriminant analysis and volcano plots were generated upon Benjamini-Hochberg correction (p ≤ 0.05).

### 2.10 Statistical analysis

Photosynthesis, fluorescence, SPAD and mineral content data were analyzed in a one-way ANOVA environment (SPSS p ≤ 0.05, Tukey *post-hoc*). Biometric data and yield were analyzed via unpaired t-test using (Excel). Metabolomics statistics were performed in Compound Discoverer 3.3 (Thermo Fisher Scientific, San José, CA) with Benjamini-Hochberg correction (p ≤ 0.05).

## 3 Results

### 3.1 Daily light integral

The experiment was conducted on potato plants placed in a cold greenhouse under full sunlight light (CTRL) or shaded but with integration lighting (LED), as described in the experimental section. During the 78 days of the experiment, some fluctuations related to seasonal conditions were observed in climatic parameters inside the greenhouse (data not shown). The mean air temperature was 15.6 °C (min: 9.6 °C; max: 30.1 °C), while air relative humidity was 81.0% (min: 52.4%; max: 98.9%).

**Table 1** reports the values of Daily Light Integral (DLI) recorded in the CTRL and LED conditions at different phenological stages of potato plants cultivated in greenhouse. As expected, the DLI value over the entire experimental period (72 DAT) was significantly lower in the LED treatment compared to CTRL, showing a 20.5% reduction. The interaction between the lighting treatment and the plant phenological stage revealed that, during vegetative growth and flowering, DLI values were comparable between the two light treatments, while during tuber bulking the LED treatment received a 36.2% less light than CTRL (**Table 1**).

**Table 1:** Daily Light Integral (DLI) recorded under natural light (CTRL) and LED-supplemented natural light (LED) at different phenological stages of potato plants.

		DLI (mol m^-2^ d^-1^)
Lighting treatment (L)	CTRL	10.37 ± 0.99 a
	LED	8.24 ± 0.45 b
Phenological stage (D)	Vegetative	6.53 ± 0.26 b
	Flowering	9.90 ± 0.57 a
	Tuber bulking	11.48 ± 1.15 a
LxD	Vegetative x CTRL	6.32 ± 0.27 c
	Vegetative x LED	6.73 ± 0.47 c
	Flowering x CTRL	10.77 ± 0.69 ab
	Flowering x LED	9.02 ± 0.76 bc
	Tuber bulking x CTRL	14.02 ± 1.52 a
	Tuber bulking x LED	8.95 ± 0.66 bc
Significance	Treatment (L)	***
	Phenological stage (D)	***
	L x D	**

Different letters indicate significant differences according to Tukey’s multiple-range test (p<0.05). **p<0.01; ***p<0.001.

Mean values ± standard errors.

In the LED treatment, the contribution of artificial light to the total DLI was constant across the stages, providing 2.05 ± 0.29 mol m^-^² d^-^¹, 1.17 ± 0.05 mol m^-^² d^-^¹, and 1.3 ± 0.19 mol m^-^² d^-^¹ during vegetative growth, flowering, and tuber bulking, respectively. On the average of the whole experiment, supplemental LED lighting contributed for 1.51 ± 0.15 mol m^-^² d^-^¹, corresponding to 18% of the total DLI.

### 3.2 Plant physiology and stomatal traits

**Figure 1:**
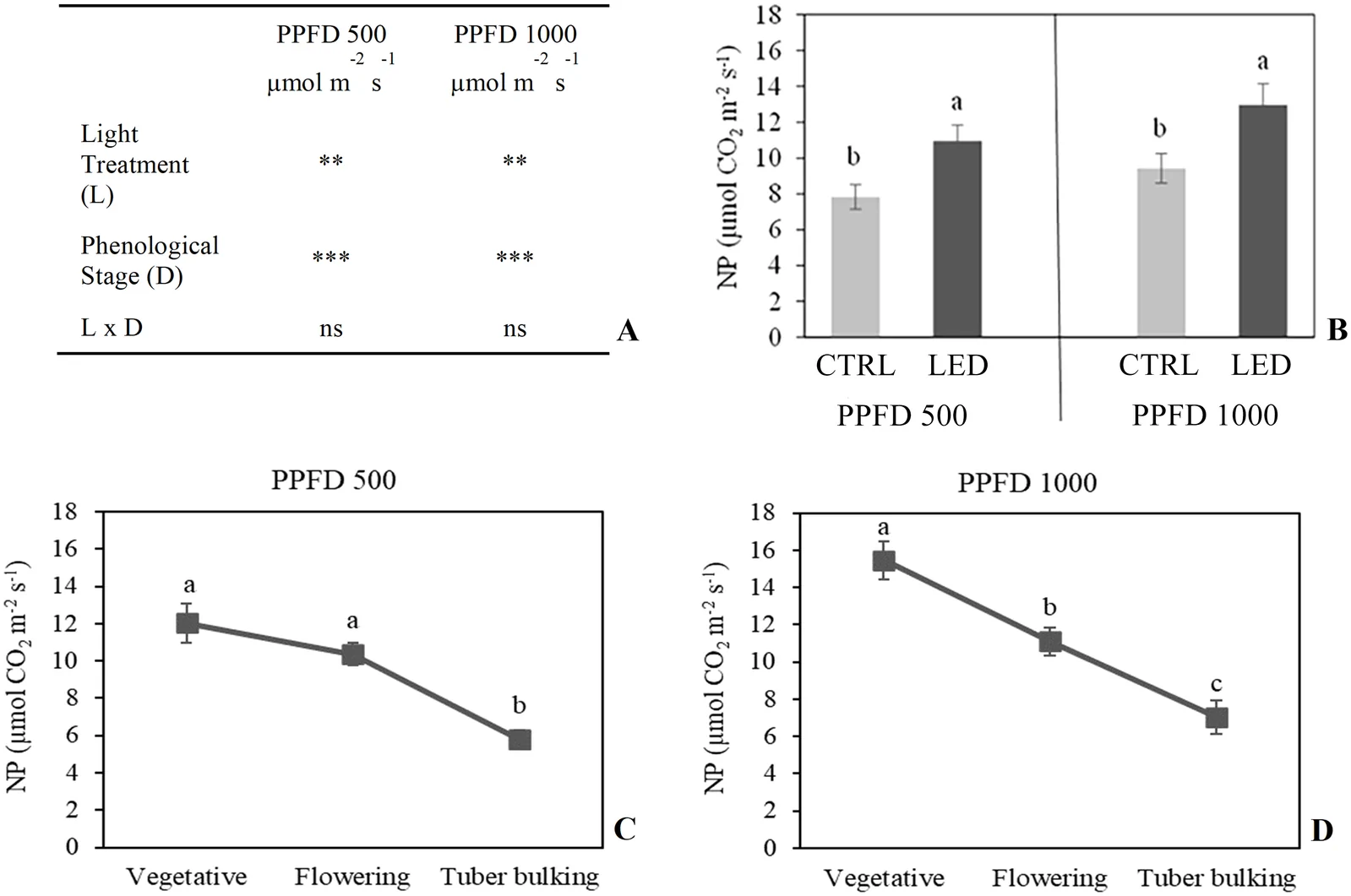
Net photosynthesis (NP) measured at different phenological stages under sub-saturating (PPFD 500) and saturating (PPFD 1000) light intensities, corresponding to 500 and 1000 µmol s^-^¹ m^-^², in potato plants cv. ‘Colomba’ grown natural light (CTRL) and LED-supplemented natural light (LED). Image **(A)** shows the statistical analysis; Image **(B)** shows a comparison of the two light treatments; Image **(C)** shows net photosynthesis at 500 PPF; and Image **(D)** shows net photosynthesis at 1000PPF. Mean values ± standard error, n = 6. ns, **, *** indicate non-significant or significant at P ≤ 0.01, and 0.001, respectively. Different letters indicate significant differences according to Tukey’s multiple-range test at p < 0.05.

**Figure 1** shows the values of net photosynthesis (NP) measured at the sub-saturating (500 µmol s^-1^ m^2^) and saturating (1000 µmol s^-1^ m^2^) irradiance for potato. Photosynthetic activity was significantly influenced by both the light treatment and the developmental stage, while their interaction was not significant (**Figure 1A**). Under LED supplementation, the NP value was significantly higher than in control, with increases of 40% and 38% at sub-saturating and saturating irradiance, respectively (**Figure 1B**). The rate of NP changed along with the plant developmental stages, progressively declining from the vegetative stage to tuber bulking, under both the light treatments and the tested irradiances (**Figures 1C, D**).

**Figure 2:**
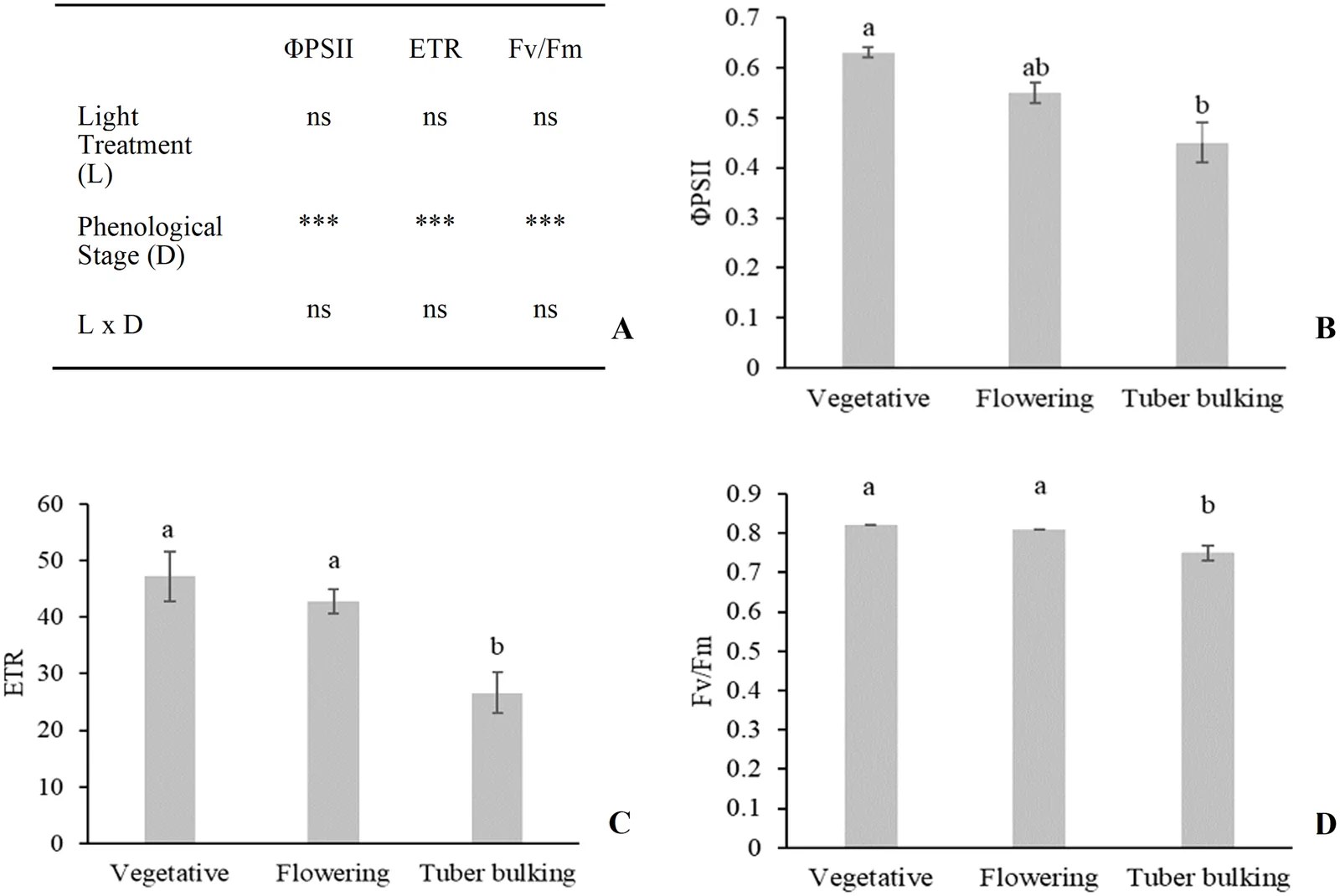
PSII quantum yield of linear electron transport (ΦPSII), electron transport rate (ETR), and maximum quantum efficiency of PSII photochemistry (Fv/Fm), measured at different phenological stages in potato plants cv. ‘Colomba’ grown under natural light (CTRL) and LED-supplemented natural light (LED) (average effect of D factor). Image **(A)** shows the statistical analysis, while Images **(B–D)** show the trends for ϕPSII, ETR, and Fv/Fm, respectively. Mean values ± standard error, n = 6. ns and *** indicate non-significant or significant at P ≤ 0.001, respectively. Different letters indicate significant differences according to Tukey’s multiple-range test at p < 0.05.

Chlorophyll fluorescence parameters, including quantum yield of PSII electron transport (ΦPSII), linear electron transport rate (ETR), and maximal PSII photochemical efficiency (Fv/Fm), were significantly affected by the phenological stage but not by the lighting treatment. These parameters exhibited a marked decline during tuber bulking, irrespective of light conditions. Since the interaction between lighting treatment and developmental stage (L × D) was not statistically significant, only the main effect of developmental stage (D), averaged across light treatments, is presented in **Figure 2**.

Leaf greenness (SPAD index) was unaffected by lighting treatment while significantly varied with phenological stage and its interaction with light conditions. Overall, the SPAD index decreased gradually from flowering to tuber bulking stage. However, at the bulking stage, a significant reduction in this parameter was observed in CTRL plants compared to LED counterparts (**Supplementary Figure 1**).

Regarding stomatal features, LED treatment slightly increased stomatal density (97.64 ± 5.72 n° mm**^-^²**) compared to the control (86.10 ± 4.25 n° mm^-^²), although the difference was not statistically significant. The stomatal size, expressed as guard cell dimensions, was also unaffected by light conditions (data not shown).

### 3.3 Plant growth, tuber yield, and tuber mineral content

**Table 2** summarizes the effect of light treatments on the main biometric parameters of both the aerial and the hypogeous parts of potato plants. Light integration with LEDs significantly enhanced all major shoot traits, with leaf area, number of leaves, and specific leaf area increasing by 64%, 30%, and 27%, respectively, compared to CTRL. Accordingly, the dry weight of leaves and stems increased by 88% and 58%, leading to an almost two-fold rise in total shoot biomass under LED treatment.

**Table 2:** Main biometric parameters of aerial and hypogeous parts of potato plants cv.

Treatment	Leaf area	Number of leaves	Specific leaf area	Leaves dry weight	Stems dry weight	Total aerial dry biomass
	(cm^2^ plant^-1^)	(n plant^-1^)	(cm^2^ leaf^-1^)	(g plant^-1^)	(g plant^-1^)	(g plant^-1^)
Aerial part						
CTRL	1321 ± 74	18.6 ± 0.8	71.14 ± 1.56	3.31 ± 0.16	1.43 ± 0.16	4.74 ± 0.31
LED	2171 ± 158	24.1 ± 1.1	90.13 ± 5.87	6.21 ± 0.26	2.27 ± 0.14	8.48 ± 0.40
	**	*	*	***	*	**
	Tuber yield	Number of tubers	Tuber fresh weight	Tubers dry weight	Roots dry weight	Total hypogeous dry biomass
	(g plant^-1^)	(n plant^-1^)	(g tuber^-1^)	(g plant^-1^)	(g plant^-1^)	(g plant^-1^)
Hypogeous part						
CTRL	227 ± 9	14.2 ± 1.1	16.28 ± 1.85	38.6 ± 1.5	2.70 ± 0.08	41.35 ± 1.48
LED	292 ± 10	17.4 ± 1.9	17.17 ± 2.06	46.1 ± 2.2	2.91 ± 0.18	48.96 ± 2.03
	**	ns	ns	ns	ns	*

‘Colomba’ grown under natural light (CTRL) and LED-supplemented natural light (LED). Mean ± standard error, n = 4. ns, *, **, *** indicate non-significant or significant differences at P ≤ 0.05, 0.01, and 0.001, respectively, according to the Student’s t-test.

The growth of underground organs was largely unaffected by lighting treatment. However, a tendency toward a higher number of slightly heavier tubers resulted in significantly greater tuber yield and total hypogeous dry biomass under LED supplementation compared with CTRL (+29% and +18%, respectively; **Table 2**). Tuber dry matter percentage (fresh weight basis) did not differ significantly between treatments, with values of 17.0 ± 0.07% in CTRL and 15.9 ± 0.47% in LED (mean ± S.E.); these data are reported here to complement **Table 2**.

The dry matter partitioning in the different plant organs revealed a lower harvest index in LED-treated plants (80.3%) compared to CTRL (83.8%), indicating a modest shift in biomass allocation from edible parts to vegetative tissues under supplemental lighting (**Supplementary Figure 2**)

As shown in **Table 3**, the mineral composition of tubers was minimally affected by light conditions. LED treatment significantly increased the nitrogen (+20%) and decreased boron (-10%) content, while the total mineral content rose by just 1.2% compared to CTRL. Potassium was the predominant nutrient, followed by nitrogen, phosphorus, sulphur, and magnesium. Minor elements such as calcium, sodium, iron, manganese, zinc, boron, copper, and molybdenum were present at ≤ 0.1 g kg^-1^ DW.

**Table 3:** Tuber mineral profile (g kg^-^¹ or mg kg^-^¹ DW) of potato cv.

Minerals	CTRL	LED	Significance
(g kg^-1^ DW)			
K	26.6 ± 0.2	25.4 ± 0.8	ns
N	9.8 ± 0.2	11.8 ± 0.6	*
P	4.1 ± 0.1	4.0 ± 0.2	ns
S	2.2 ± 0.1	2.0 ± 0.1	ns
Mg	1.3 ± 0.1	1.3 ± 0.1	ns
(mg kg^-1^ DW)			
Ca	119.5 ± 10.7	106.8 ± 14.9	ns
Na	48.3 ± 2.0	49.6 ± 2.8	ns
Fe	27.2 ± 2.2	22.6 ± 1.2	ns
Mn	10.9 ± 1.1	10.8 ± 0.8	ns
Zn	8.1 ± 0.2	9.0 ± 0.6	ns
B	6.4 ± 0.1	5.8 ± 0.2	*
Cu	5.3 ± 0.2	4.9 ± 0.6	ns
Mo	1.6 ± 0.1	1.5 ± 0.1	ns

‘Colomba’ grown under natural light (CTRL) and LED-supplemented natural light (LED). Mean values ± standard errors; n = 6. ns, *, non-significant or significant at P ≤ 0.05, respectively, according to the Student’s t-test.

### 3.4 Transcriptomic reveals key changes related to carbohydrate metabolism and stress-responsive genes after plant LED lighting

**Figure 3:**
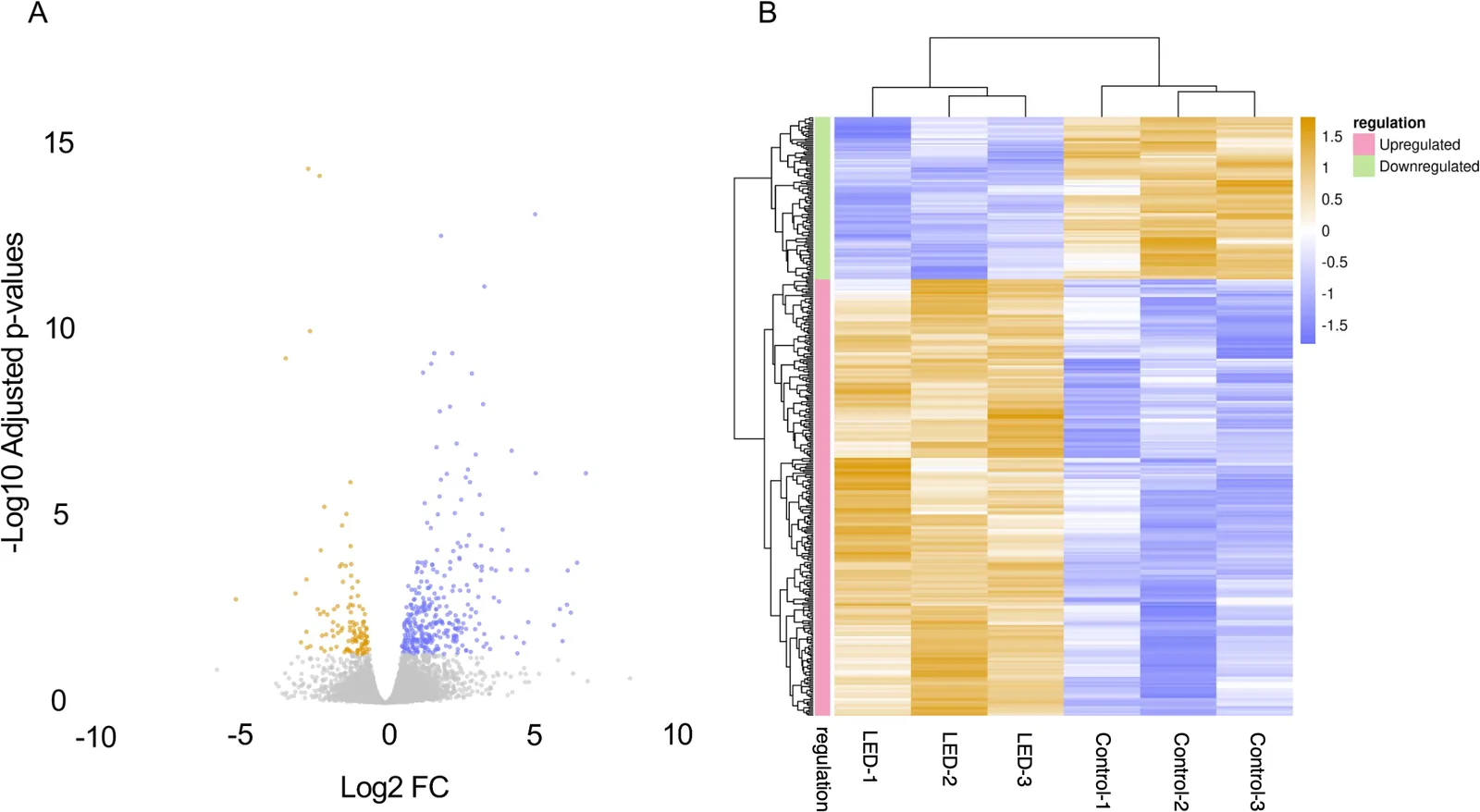
Transcriptomic analysis of *S. tuberosum* under LED and CTRL conditions. Differential expression analysis comparing plants grown under red-blue LED light and natural sunlight. **(A)** Volcano plot of DEGs showing log_2_ fold change vs. –log_1__0_ adjusted p-value. Orange and purple dots indicate up-regulated and down-regulated genes, respectively, while grey ones are non-significant. **(B)** Heatmap of hierarchical clustering of DEGs, with columns representing biological replicates and rows representing genes. Z-score color gradient: orange for up-regulated, blue for down-regulated genes.

RNA-seq analysis identified 442 differentially expressed genes (DEGs) between *S. tuberosum* tubers from plants exposed to LED lighting and natural sunlight, including 329 up-regulated and 113 down-regulated transcripts (FDR < 0.05) (**Figure 3A**, **Supplementary Table 3**). Hierarchical clustering confirmed a neat separation between treatments (**Figure 3B**), with LED exposure associated with the up-regulation of chlorophyll a/b-binding proteins, as well as genes involved in polyamine metabolism, such as those encoding putative gamma-glutamyl-gamma-aminobutyrate hydrolase, and volatile terpenoid biosynthesis, including R-linalool synthase (**Table 4**). GO and KEGG enrichment analyses revealed significant over-representation of biological processes related to photosynthesis, light harvesting, chlorophyll biosynthesis, redox homeostasis, carbon fixation, and pigment metabolism (**Supplementary Tables 4** and **5**; **Supplementary Figure 3**). Conversely, down-regulated genes were mainly associated with nucleosome organization (GO) and protein processing in the endoplasmic reticulum (KEGG).

**Table 4:** Top-10 differentially expressed genes (DEGs) in *S. tuberosum* tubers grown under LED and control (natural sunlight) conditions.

Gene ID	Gene description	log2 fold change	Adjusted p-value (FDR)	Regulation
Soltu.DM.02G014010	chlorophyll a/b binding protein	6.9106	7.188e-07	Up-regulated
Soltu.DM.07G017880	genomic DNA, chromosome 3, P1 clone: MSD21	6.6079	0.000178	Up-regulated
Soltu.DM.03G015580	–	6.3922	0.0039	Up-regulated
Soltu.DM.03G000900	chlorophyll a/b binding protein//chlorophyll a-b binding protein 1, chloroplastic-related	6.2863	0.00029	Up-regulated
Soltu.DM.02G013810	chlorophyll a/b binding protein//subfamily not named	6.2599	0.0024	Up-regulated
Soltu.DM.03G036990	Leucine Rich Repeat (LRR_1)//Protein tyrosine kinase (Pkinase_Tyr)//Leucine rich repeat N-terminal domain (LRRNT_2)	6.1068	0.0224	Up-regulated
Soltu.DM.02G014020	chlorophyll a/b binding protein//subfamily not named	6.0141	0.0031	Up-regulated
Soltu.DM.01G045180	R-linalool synthase/(3R)-linalool synthase	5.8110	0.0083	Up-regulated
Soltu.DM.02G014040	chlorophyll a/b binding protein//subfamily not named	5.1832	7.1885e-07	Up-regulated
Soltu.DM.03G004610	Gamma-glutamyl-gamma-aminobutyrate hydrolase/Gamma-glutamyl-GABA hydrolase	5.1637	8.43212e-14	Up-regulated

Listed genes represent those with the highest absolute log_2_ fold change and an adjusted p-value (FDR) < 0.05. Functional annotations include chlorophyll-binding proteins, secondary metabolism enzymes, and signaling-related domains.

Several DEGs associated with sterol and brassinosteroid (BR) biosynthesis were differentially expressed (**Supplementary Table 3**), including down-regulated putative *sterol 24-C-methyltransferase* homologs (SMT1/SMT2) and up-regulated genes annotated as *7-dehydrocholesterol reductase* (DWF5), *cytochrome P450 CYP710A1*-like enzymes, and *UDP-glucosyltransferases* of the UGT73C subfamily. These changes suggest a shift in sterol profiles and BR conjugation under LED treatment. Genes encoding enzymes involved in carbohydrate metabolism, including *sucrose-phosphate synthase* (SPS1/SPS3), *sucrose synthase* (SUS), *glyceraldehyde-3-phosphate dehydrogenase* (GAPA1), *glucose-6-phosphate dehydrogenase* (G6PDH), and *triosephosphate isomerase* (TPI), were significantly up-regulated, indicating enhanced sugar turnover and carbon flux through glycolysis, the Calvin cycle, and the oxidative pentose phosphate pathway (**Supplementary Table 3**). Additionally, DEGs related to *trehalose-6-phosphate synthase* (TPS1), *raffinose synthase* (RAFS), and *phospholipase A1* (PLA1) were induced, reflecting coordinated adjustments in sugar signaling, osmoprotection, and lipid remodeling.

In parallel, several stress-responsive genes were down-regulated under LED lighting, including homologs of *small heat shock proteins* (HSP17.6C-CI, HSP17.6A), *HSP70*, *HSP90-1*, *thioredoxin H-type 1* (TRXh1), and the *tonoplast intrinsic protein TIP2-1*, consistent with a globally attenuated stress response in LED-grown plants (**Supplementary Table 3**). carbon fixation, and pigment metabolism.

### 3.5 Proteomics unveils differentially represented proteins involved in stress response, TCA cycle, and biosynthesis of secondary metabolites after plant LED lighting

**Figure 4:**
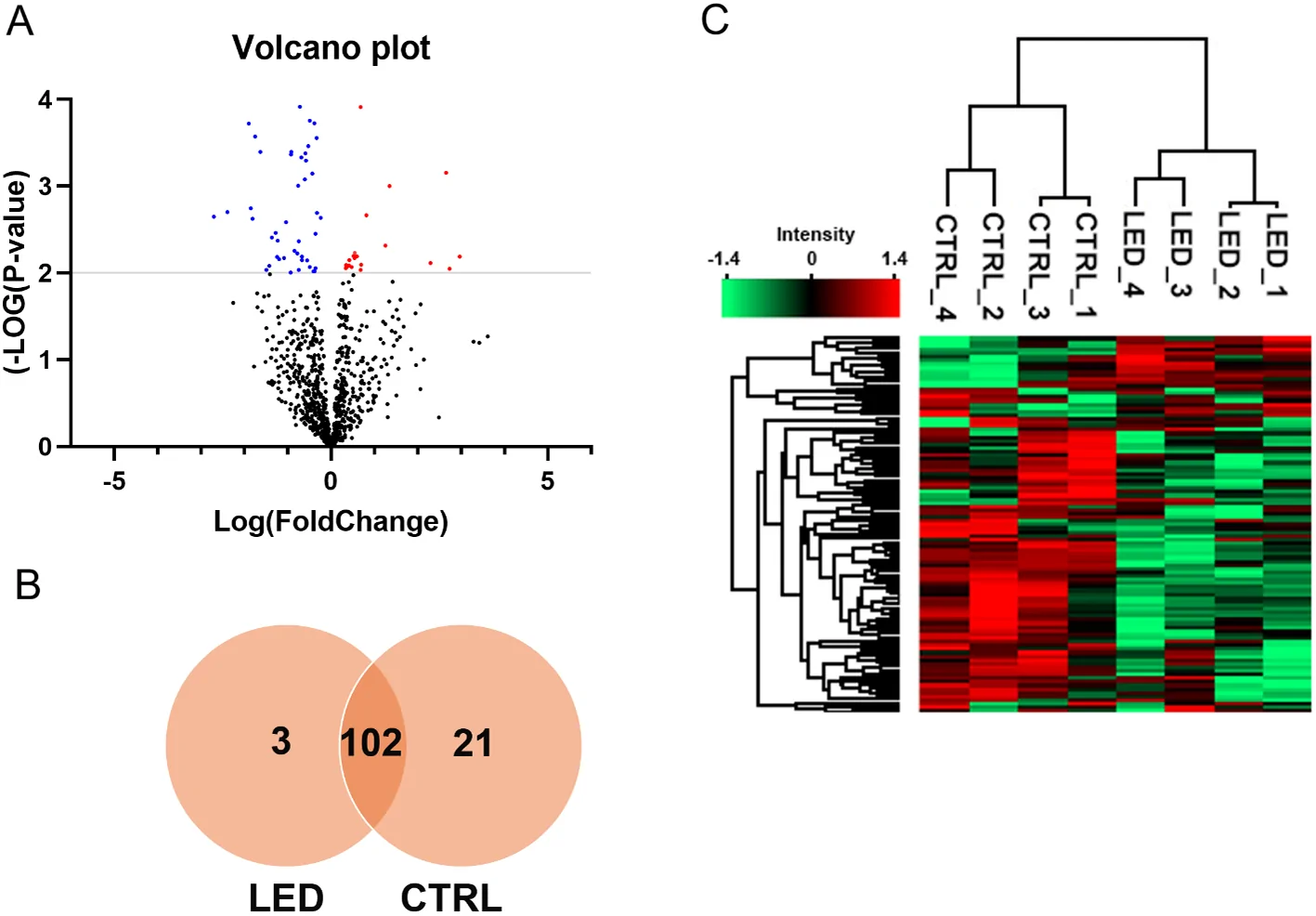
**(A)** Volcano plot of differentially represented proteins (DRPs). This figure displays the distribution of protein representation changes between LED-treated and CTRL samples. Specifically, the spots colored in red and blue are associated with the over-represented or down-represented proteins, respectively. Conversely, the spots reported in black correspond to proteins not showing significant quantitative variations between the two experimental conditions. **(B)** Venn diagram illustrating the distribution of DRPs across treatments. **(C)** Heatmap of hierarchical clustering of DRGs, with columns representing biological replicates of LED-treated and CTRL samples, and rows representing variably represented proteins between the two experimental conditions. Z-score color gradient: red for over-represented and green for down-represented proteins. The intensity value depicts the direction of protein representation.

**Figure 5:**
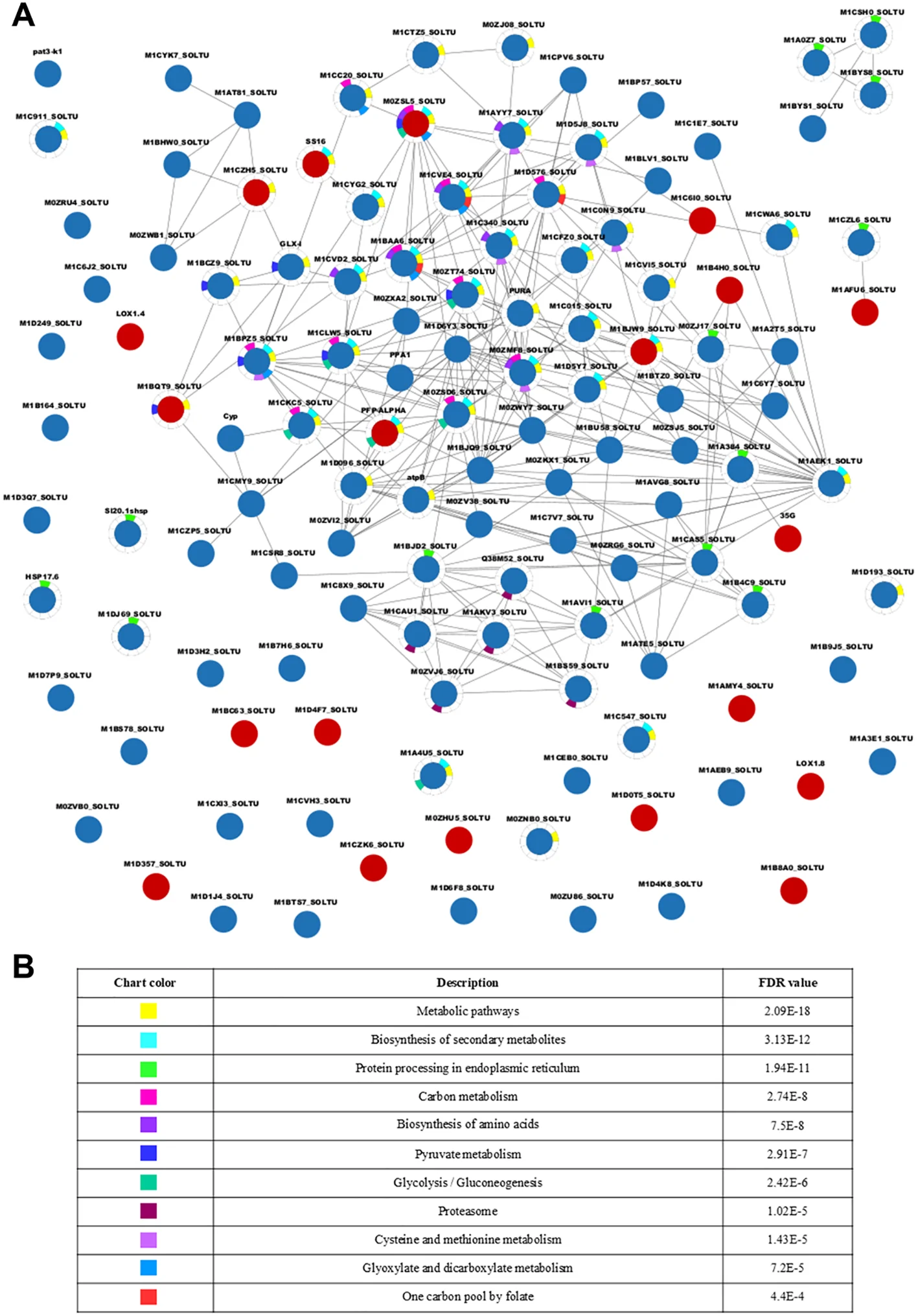
STRING analysis of differentially represented proteins (DRPs) between LED-treated and CTRL samples. **(A)** Protein interaction network of DRPs (126 in number) identified between the two experimental conditions. Specifically, the spots colored in red and blue are associated with the over-represented or down-represented proteins, respectively. Additional colors refer to functional annotation as reported in panel **(B)** Medium-confidence interactions (0.4) are shown. For annotation codes and corresponding protein names, please refer to **Supplementary Table 6**. **(B)** Functional analysis of DRPs identified in LED-treated and CTRL samples. Differentially represented proteins (126 in number) were subjected to functional enrichment through STRING suite based on KEGG pathway database. The top 11 pathways are reported. Shown is the pathway code, the corresponding description, and false discovery rate (FDR).

A total of 860 proteins were identified; after filtering data to exclude contaminants, reverse sequences, and proteins with fewer than three valid LFQ values, 126 proteins were retained as differentially represented (DRPs; p < 0.05) based on a label-free quantification (LFQ) approach (**Supplementary Table 4**). These DRPs were evenly distributed between over- (20 in number) and down-represented (106 in number) proteins in LED-treated samples, as shown in the volcano plot and heatmap (**Figures 4A, C**). A Venn diagram (**Figure 4B**) illustrates that 102 DRPs were shared between LED-treated and CTRL samples, while 3 were unique to the LED group, and 21 to the control. All above-reported DRPs were subjected to bioinformatic analysis with STRING suite, showing that a significant part of them (79 in number) were strictly interconnected in a populated and ramified network (**Figure 5A**), while the remaining ones were included in a quaternary and a binary assembly, or did not show molecular association (41 in number). The occurrence of most DRPs in the main association network emphasized the existence of a functional assembly bridging components from various deregulated metabolic pathways/molecular processes associated with plant LED irradiation.

Functional enrichment analysis performed with KEGG through STRING revealed that DRPs were mostly associated with general metabolic pathways, biosynthesis of secondary metabolites and amino acids, protein processing/folding, carbon metabolism, glycolysis/gluconeogenesis, proteasome, as well as pyruvate, cysteine/methionine and glyoxylate/dicarboxylate metabolism (**Figure 5B**). STRING-mediated Gene Ontology (GO) analysis of over- and down-represented proteins conducted for biological processes further supported and integrated these findings (**Supplementary Figures 4A, B**). Notably, various stress-related proteins, including heat shock proteins (M1A0Z7, M1AJ64, K7VKA6, M1CZL5, M1BYS8, M1CSH0, M1DJ69, M1B4C9, M1A2T5, M1CZL6), polypeptide folding assisting components (M1ATE5, M1CZP5, M1CSR8, M0ZJ17, M1C7V6, M1A383, M1AVG8, Q9XF12), ROS-scavenging enzymes (M1CMY9, M0ZXA4, M1C911, M1CVI5, M0ZNB0, M1AE74, M1BCZ9, M1BLV1) and pathogen/insect response components (M1BS78, M1D4K8, Q3YJS9) were down-represented in LED-treated plants, suggesting a less stressful environment under this condition. On the other hand, pyruvate kinase (M1CLW5) and pyruvate dehydrogenase (M0ZT74) were exclusively detected in control samples, while phosphopyruvate hydratase (M0ZSD6) was down-represented in LED-treated plants, suggesting a reduced metabolic flux toward acetyl-CoA under artificial lighting conditions, and consequently to anabolic pathways leading to fatty acids, steroids and glycoalkaloids. In the latter context, significant were also the observed reduced levels of acetyl-CoA C-acyltransferase (M1CWA6), isopentenyl-diphosphate Δ-isomerase (M1C547), and a member of the 2OG-Fe(II) oxygenase family (M0ZVB0). Moreover, key enzymes like tryptophan synthase (M1CVD2), formamidase (M1CC20), N-acetyl-gamma-glutamyl-phosphate reductase (M1CFZ0) and beta-ureidopropionase (M1CTZ5) were significantly down-represented in LED samples, indicating a reduced capacity to recover nitrogen to yield N-containing defense compounds, including glycoalkaloids. Similarly, various enzymes involved in metabolic pathways associated with methionine, cysteine, glycine, serine, glutamic acid and aspartic acid were also down-represented in LED-treated plants. Accordingly, functional mapping highlighted the link between central carbon metabolism and secondary metabolites biosynthesis, underscoring a metabolic shift favoring plant growth over defense (**Figure 5B**; **Supplementary Figure 4A, B**).

Conversely, the limited number of proteins over-represented under LED supplementation were mainly associated with carbohydrate turnover (sucrose synthase M0ZT40 and pyrophosphate-fructose 6-phosphate 1-phosphotransferase M1CV13), energy metabolism (NADH dehydrogenase M1CZH5), lipid biosynthesis and membrane remodeling (acyl-[acyl-carrier-protein] desaturase M1BC63, phospholipase A1 M1D4F7, and two lipoxygenases O22508 and Q43190), and signaling (protein tyrosine phosphatase M1B8A0 and 14-3–3 protein P93787). Notably, despite the observed reduction of stress-related proteins, osmotin (M0ZHU5), a PR-5 family component associated with abiotic/biotic stress defense, and polygalacturonase inhibiting protein (M1AFU6) inhibiting pectin-degrading enzymes from fungal and bacterial pathogens, were highly represented in LED-treated samples.

A concordant modulation between transcriptomic and proteomic datasets was detected in LED-treated plants. Transcripts encoding key enzymes involved in carbohydrate metabolism, including *SPS1/SPS3, SUS, GAPA1, G6PDH*, and *TPI*, were significantly up-regulated, consistent with the increased abundance of sucrose synthase (M0ZT40) observed at the protein level. Similarly, NADH dehydrogenase (M1CZH5) was over-represented at both transcript and protein levels. In contrast, several stress-associated genes, including *HSPs, TRXh1*, and *TIP2-1*, were down-regulated under LED treatment, paralleling the reduced abundance of corresponding stress-related proteins such as heat shock proteins (M1A0Z7, M1AJ64, K7VKA6) and ROS-scavenging enzymes (M1CMY9, M1C911).

Transcriptomic data also hinted at a potential suppression of the plant’s chemical defense system. This was confirmed by proteomic evidence showing the widespread down-representation of crucial enzymes involved in biotic/biotic stresses and their redox mediators. Moreover, significant quantitative changes in transcripts and proteins associated with the biosynthesis of precursors of specific secondary metabolites were also observed, including glycoalkaloids. In some cases, the direct correlation observed between decreased gene expression and reduced protein abundance for these enzymes signified a lowered capacity for producing defense compounds. In essence, both transcriptomic and proteomic datasets converged to paint a consistent picture: LED lighting drives a metabolic shift in *S. tuberosum* that redirects resources from defense mechanisms towards plant growth and production. While transcriptomics highlighted the potential for change through gene expression, proteomics provided direct evidence of functional shifts at the molecular machinery level, confirming the actual changes in protein abundance and activity that underpin this metabolic reprogramming. Overall, the integration of transcriptomic and proteomic data provided complementary insights into plant responses to LED lighting.

### 3.6 Untargeted metabolomics describes primary and secondary metabolite variations due to LED lighting

**Figure 6:**
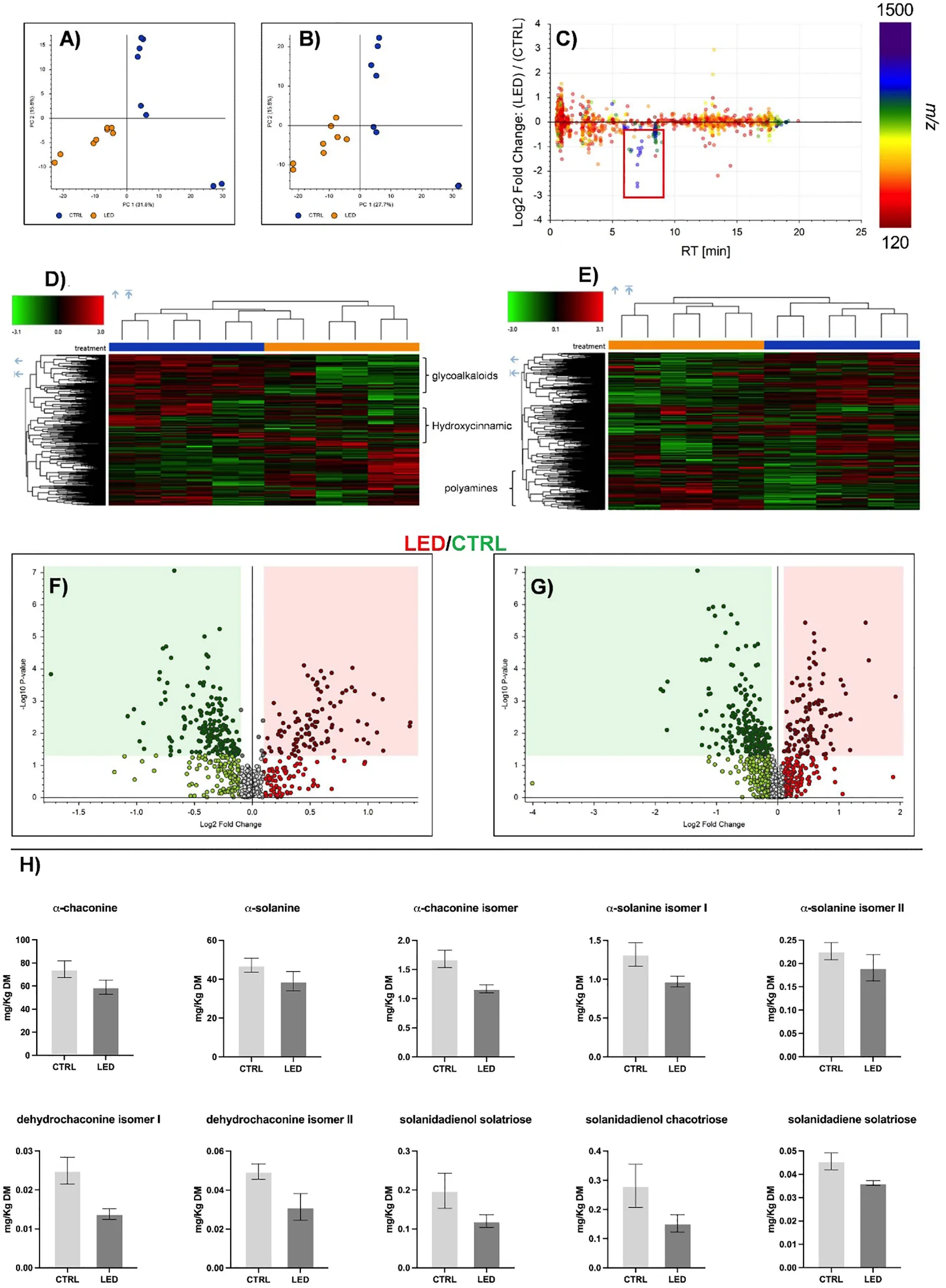
Untargeted metabolomic and multivariate data analysis revels a determinant role for glycoalkaloids, hydroxycinnamic acids, amino acids and other secondary metabolites in potato under CTRL and LED conditions. **(A)** PCA score plot of metabolites in CTRL samples (blue points) and LED (orange points) samples in negative ion mode explaining 47.6% and **(B)** positive ions explaining 43.5% of the variability considering the first two components on x and y axes, respectively. **(C)** 3D plot LC–MS/MS analysis in negative ion mode in the time range 0–25 min (x axis). The y axis reports the log2 fold change of the LED condition over the control counterpart; z axis summarizes the *m/z* value of each feature ranging from red (*m/z* 120) to purple (*m/z* 1500) representative of screened signals upon negative and positive data analysis workflows over blank procedural samples. See supplementary **Supplementary Figure 5** for the corresponding positive ion mode plot and supplementary **Supplementary Table 7** for an overall picture of the differences between over- and under-represented metabolites. **(D)** Heatmap hierarchical cluster analysis of metabolites detected in negative ion mode reporting in orange LED samples and in blue CTRL samples. **(E)** Heatmap hierarchical cluster analysis of metabolites detected in positive ion mode with coloring as in panel **(D)** Dendrograms on the left of panels **(D, E)** report the molecular grouping and distance between molecular classes, while dendrograms on their top report the sample grouping. Normalized area ranges and Euclidean distance were used in all cases; scaled expression values of each range are plotted in green to red through black color scale. **(F)** Volcano plot in negative ion mode depicts the compound area counts in LED and CTRL samples through a 1 versus 1 comparison by using the ratio LED/CTRL; features in the green region report over-represented analytes in CTRL with a Log2 mean ratio fold-change value lower than −0.1 and a Log10 for a P-value higher than 1.5. False discovery rate correction was applied using the Benjamini–Hochberg *post-hoc* analysis, and a significance level of 0.05 was used. **(G)** Volcano plot for positive ionization mode with coloring as in panel **(F)**. **(H)** Key glycoalkaloids identified and quantified in the tuber of potato grown in CTRL and LED conditions. The figure shows the concentration of various glycoalkaloids in LED and CTRL samples (n=4).

To identify the metabolomic signature of the tubers in LED and CTRL conditions, we used a consequential workflow starting with metabolite annotation (alternatively in positive and negative ion mode), then identification and quantification by means of pure reference standards (**Supplementary Table 5**). **Figure 6** summarizes the main results regarding metabolite annotation and identification; upon background removal, filtered compounds were loaded into the principal component analysis (PCA) to explore the sample distribution over a 2D space. PCA plot for negative (**Figure 6A**) and positive ions (**Figure 6B**) explained 47.6 and 43.5% of the total variance, respectively, considering the first two components with a distinct separation between LED and CTRL samples. The sample clustering indicated that the lighting treatment was the main factor contributing to the observed variability in metabolite production modulation. To include analytical information on the different phytochemicals, we further explored the qualitative and semi-quantitative response in negative ion mode through a three-dimensional plot (**Figure 6C**) of retention time, Log2-fold change (LED/CTRL), and *m/z* values. Analyte responses exhibited a nearly symmetrical distribution centered around the zero value over time along the y-axis, except during the retention time interval from 2 to 9 min, where negative values of Log2 fold change suggested a down-representation of metabolites in LED samples. The use of a color gradient from red to purple in the multivariate data analysis underscored the metabolic complexity and response variability in potato plants exposed to different lighting treatments. Indeed, blue-labelled compounds emerged in the above-reported retention time interval, representing *m/z* values around 1000, indicative of compounds with higher molecular weight accumulated in the standard conditions. 3D plot distribution in positive ion mode paralleled the results in negative ion mode (**Figure 6C**). Then, two interrelated heatmaps based on hierarchical clustering analysis (HCA) of detected metabolites in negative ion modes interpolated sample grouping and molecular pattern associated with the two treatments (color scale of normalized areas ranging from green, low response, through black to red, high response; **Figures 6D, E**).

In negative ion mode HCA, we spotted an intense red region in the top left corner, constantly over-represented in control samples and including most of the compounds belonging to the glycoalkaloid families. To further sieve the most significant changes in metabolites between LED and CTRL samples, a discriminant analysis was performed and data were reported through a volcano plot through a 1 versus 1 comparison by using the ratio LED/CTRL (**Figure 6F** shows the negative ion mode volcano plot with 95 compounds over-represented in LED treatment, red region and red labelled compounds), while 169 compounds were down-represented (hence over-represented in CTRL samples, green region with green-labelled compounds).

In parallel, volcano plot in positive ion mode spotted 115 analytes significantly over-represented in LED samples and 177 down-represented when compared with CTRL samples (green region with green-labelled compounds, **Figure 6G**). To collect all the information obtained in the metabolomic analysis, regardless of the ionization mode, we decided to focus on the three classes pinpointed by the overall multivariate data analysis, including polyphenols, polyamines, amino acids and their derivatives, glycoalkaloids, organic acids, sugars and others (**Supplementary Table 5**). Glycoalkaloids, polyphenols and most of the amino acids and their derivatives showed lower concentrations in tuber grown under LED lighting compared to those in sunlight. The most abundant glycoalkaloids consisted of α-solanine and α-chaconine, followed by dehydrochaconine, solanidadienol solatriose, solanidadienol chacotriose, and two isomers of solanidadiene solatriose (**Figure 6H**). The reduced concentration of these glycoalkaloids in LED samples paralleled the decrease of their aromatic amino acids precursors such as tryptophan (**Supplementary Figure 5**). In line with glycoalkaloids, polyphenol members of the hydroxycinnamic acid family, as chlorogenic acid, neochlorogenic acid, cis chlorogenic acid, 4-*O*-feruloyl-D-quinic acid, 3,5-dicaffeoyl quinic acid, caffeic acid hexoside ferulic acid hexoside and 1-*O*-vanilloyl-beta-D-glucose, were down-represented in LED over CTRL samples, similarly to ascorbic acid and glutathione (**Supplementary Figure 5**). Conversely, polyamine derivatives and organic acids were significantly higher in LED samples; two key examples are *N*-caffeoylputrescine and *N-*feruloylputrescine. Biogenic amine derivatives pinpointed the accumulation of some of their precursors as arginine, glutamine, ornithine, *N,N*-dimethylarginine and L-pyroglutamic acid, all with a markedly higher response in LED condition (**Supplementary Figure 5**). Finally, among the organic acids, pipecolic acid and quinic acid were present at higher concentrations in LED samples.

## 4 Discussion

We evaluated the feasibility of vertical cultivation of potato in greenhouse using supplemental LED lighting to compensate for the sunlight reduction due to the shading from tower shelves. Despite a lower DLI, net photosynthesis significantly enhanced in the LED treatment compared to natural light. This enhancement was not found in relation to changes in stomatal density, highlighting the efficiency of RB wavelengths in sustaining plant assimilation, by matching the chlorophyll absorption peaks and improving the photosystem efficiency (Rehman et al., 2024). Particularly, blue light further promotes stomatal opening, CO_2_ uptake, and chlorophyll biosynthesis, while red light supports energy capture and canopy expansion (Bantis et al., 2018).

Photosynthetic activity peaked during the vegetative stage and declined through flowering and tuber bulking, despite the increasing DLI from January to March. This trend likely reflected a developmental shift from canopy growth to reproductive organ formation (Gregersen et al., 2014). Minor timing differences in measurement may also explain the discrepancy with previous data from Paradiso and coworkers who observed a peak of photosynthetic rate at flowering (Paradiso et al., 2019); in the present study, photosynthesis was recorded later (50 and 62 DAT vs. 27 and 55 DAT), possibly overlapping with tuber initiation and early resource reallocation (Thornton, 2020).

Leaf chlorophyll decreased with plant age but remained higher under RB lighting during the tuber bulking and the related plant senescence. This suggests that lower DLI and red-enriched light may delay chlorophyll degradation, allowing photosynthesis to persist longer. Consistently, previous studies reported delayed senescence in potato plants exposed to R light compared to W light (He et al., 2022). This delay in senescence was supported here by transcriptomic data, which outlined a strong enrichment for chlorophyll biosynthetic processes and thylakoid membrane components in LED-treated plants, highlighting that RB spectra not only maintain photosynthetic capacity but also molecularly reinforces chloroplast function and longevity. Fluorescence data supported this finding; indeed, Fv/Fm values (~0.83) during vegetative and flowering stages indicated an optimal photosynthetic apparatus efficiency (Maxwell and Johnson, 2000), declining later as energy was redirected toward the tuber formation.

Supplemental RB lighting also promoted vegetative growth, increasing leaf number and area, and dry matter accumulation of aerial parts. Higher specific leaf area under RB irradiation suggests a photomorphogenetic response: the development of thinner, broader leaves might be aimed at optimizing the plant light interception. This greater canopy capacity supported the observed increase in tuber yield, driven more by the improved carbon assimilation than by the light quantity *per se*. In line, transcriptomic data on tuber tissues of LED-treated plants revealed a strong up-regulation of genes involved in photosynthesis and carbon metabolism, including chlorophyll a/b binding proteins of photosystems I and II, and light harvesting components. KEGG enrichment pinpointed a significant activation of antenna proteins and Calvin cycle pathway, consistent with the observed increases in NP and SPAD index. These molecular changes likely underlay enhanced photosynthetic efficiency and carbon assimilation under RB lighting, despite the lower DLI. A similar quantitative trend was observed for genes related to carbohydrate metabolism and Krebs cycle, which were upregulated in irradiated plants. However, for both photosynthesis and carbohydrate metabolism, no significant coherent quantitative proteomic variations were observed in LED-treated plants, except for sucrose synthase and pyrophosphate-fructose 6-phosphate 1-phosphotransferase, confirming previous discrepancies between transcriptomic and proteomic data in eukaryotes (Griffin et al., 2002). The poor overlap of transcript and protein levels suggests that many transcriptional changes may not immediately translate into protein abundance, likely due to post-transcriptional regulation or delayed protein turnover.

Above-reported changes in biometric and physiological parameters in LED-treated plants did not correspond to significant modifications in tuber dry matter content, suggesting that the corresponding increased yield was associated with higher water content. These findings align with prior studies showing that R light enhances stem elongation and leaf expansion, promoting overall biomass (Mitchell and Stutte, 2015). In agreement, He and coworkers observed a greater tuber yield under RW light compared to BW and RBW treatments (He et al., 2020), while Rahman and colleagues found that R+B+W and natural light produced the highest yield compared to other light spectra (Rahman et al., 2024). In the present study, higher levels of polyamine derivatives, organic acids, D-(+)-xylose, acetylcholine and specific amino acids (e.g., arginine, glutamine, *N*-acetyl-L-leucine, ornithine, L-pyroglutamate, *N,N*-dimethylarginine, and dipeptides such as Val-Glu) were measured in tubers from LED-treated plants. These metabolites are typically associated with osmotic regulation and membrane stabilization in abiotic stress; accordingly, they have been reported as osmoprotective compounds (Kumar et al., 2024). Their accumulation under LED irradiation might suggest the occurrence of an adaptive metabolic shift favoring osmotic homeostasis and resilience to aging stress, especially during tuber bulking (Bolton, 2009), which maintains cell turgor and delays dehydration, thus determining the higher water content measured in the corresponding potatoes, without compromising the nutritional quality.

Interestingly, the integration of natural light with RB wavelengths reduced the harvest index, indicating a lower allocation to edible organs relative to total biomass (hence a greater quantity of plant waste). This could challenge waste management in closed systems. However, residual biomass may be repurposed for composting to enhance fertility of soils as well as of poor substrates in extreme environments, such as Martian or lunar regolith (Duri et al., 2025).

LED treatment significantly increased nitrogen concentration in tubers, suggesting improved nutritional value compared to controls. Proteomic analysis revealed a variable representation of key proteins involved in nitrogen metabolism and protein biosynthesis under LED lighting, such in the case of glutamate–tRNA ligase and asparagine–tRNA ligase essential for amino acid incorporation during translation, which showed higher levels in LED-treated plants. Quantitative metabolomic data on *N*-caffeoylputrescine, *N-*feruloylputrescine, and their precursors arginine, glutamine, ornithine, *N,N*-dimethylarginine and L-pyroglutamic acid were in line with the observed increased nitrogen concentration in LED-treated tubers. Altogether, these findings support the hypothesis that LED lighting may promote N assimilation and protein biosynthesis, confirming previous reports on other red light irradiated crops (Soufi et al., 2023) and reinforcing the role of LED lighting in optimizing tuber nutritional quality.

In BLSSs, food crops must meet stringent nutritional, safety, and acceptability standards to support long-duration missions (Douglas et al., 2020). These attributes largely depend on the chemical components of the edible tissues, including sugars, polysaccharides, proteins, lipids, minerals, vitamins, water, antinutrients, and phytochemicals (Coultate, 2023). Light quality, intensity, and photoperiod can modulate plant metabolism by activating specific biosynthetic pathways, ultimately shaping food quality, stress responses, and adaptive mechanisms (Rehman et al., 2024). Untargeted metabolomic revealed key differences in primary and secondary metabolic pathways. Tuber developed under LED light showed a significant reduction in the levels of polyphenols compared to CTRL, specifically in the case of hydroxycinnamic acid derivatives, as caffeic acid glucosides and chlorogenic acids. These findings are consistent with previous evidence indicating that low polyphenol accumulation is typical of tubers, while enzymes such as OsUGT706D1 (flavone 7-*O*-glucosyltransferase) and OsUGT707A2 (flavone 5-*O*-glucosyltransferase) are associated with stress-tolerant plants (Peng et al., 2017). Polyphenols are potent antioxidants, typically over-represented under stress to mitigate oxidative damage to membranes, proteins, and the photosynthetic apparatus (Bolat et al., 2024). Their reduced concentration in LED-treated tubers suggest a decreased oxidative or aging-related stress, consistent with the decline in PSII quantum efficiency (Fv/Fm) during bulking. Transcriptomic data further supported this interpretation, showing a general down-regulation of stress-responsive genes following LED treatment, including small HSPs (HSP20 family), HSP70, and antioxidant-related transcripts such as thioredoxin. The down-regulation of stress-responsive genes was mirrored at protein level, with ascertained decreased representation of abiotic stress-related proteins, including various heat shock proteins (10 in number), many chaperones, protein disulfide and prolyl *cis-trans* isomerases assisting polypeptide folding (8 in number), and ROS-scavenging enzymes (8 in number) including different superoxide dismutases, peroxidases and proteins involved in glutathione metabolism. In few cases, a coherent quantitative trend of specific transcript and protein levels was observed. These findings were paralleled with coherent reduced levels of antioxidant ascorbic acid and glutathione in LED-treated plants. Altogether, these findings support the hypothesis of a lower oxidative burden or more efficient stress mitigation under RB irradiation, emphasizing an efficient physiological adaptation of potato plants to artificial lighting. Conversely, potato plants exposed to natural sunlight have been reported to activate the shikimate/phenylpropanoid pathway, producing increased levels of phenolic acids, including various chlorogenic acid isomers, feruloyl-quinic acid derivatives, and vanillic acid conjugates (Wang et al., 2024). Transcription factor bHLH1 was shown to regulate StCCoAOMT, a key enzyme converting caffeoyl-CoA to feruloyl-CoA, a central step in phenolic acid biosynthesis (Wang et al., 2024). At present, the effect of RB LED light on the expression of *bHLH1* and *StCCoAOMT* genes remains unknown.

LED-grown tubers showed lower levels of glycoalkaloids, notably α-solanine, α-chaconine and their derivatives, which contribute to plant defense but can be toxic at high concentrations for human ingestion (Schrenk et al., 2020). Biosynthesis of glycoalkaloids is generally genotype-dependent, and light intensity and quality can influence metabolite levels (Singh et al., 2020). For instance, Nitithamyong and coworkers reported an increased glycoalkaloid content with higher light intensity (Nitithamyong et al., 1999), while Paradiso and colleagues noted that RB light alters the α-chaconine to α-solanine ratio in a cultivar-specific-manner (Paradiso et al., 2019). More recently, Okamoto and coworkers demonstrated that both red and blue light activate glycoalkaloid biosynthesis genes during post-harvest storage (Okamoto et al., 2020). Our results confirmed α-solanine and α-chaconine as the dominant glycoalkaloids in potato tuber, coherently with existing literature (Razgonova et al., 2023), and provided additional information on quantitative levels of these glycoalkaloids and their derivatives following RB LED irradiation.

From a nutritional perspective, polyamines are gaining recognition for their health benefits, particularly for anti-inflammatory and urological applications (Qiao et al., 2024). Their biosynthesis begins with the decarboxylation of ornithine or arginine to putrescine, which is further converted to spermidine and spermine (Kumar et al., 2024). The elevated levels of arginine and ornithine under LED conditions suggest a metabolic redirection toward polyamine biosynthesis. This metabolic shift was corroborated at the transcriptomic level by the observed up-regulation of genes associated with polyamine biosynthesis, such as gamma-glutamyl-gamma-aminobutyrate hydrolase. Together, these molecular and metabolic data suggest that LED-treated plants activate biosynthetic pathways during tuber bulking favoring augmented production of these health beneficial compounds. Interestingly, asparagine levels were lower in LED-treated tubers, which is advantageous for food safety. Asparagine is the key precursor of acrylamide, a potentially carcinogenic compound formed during high-temperature cooking via the Maillard reaction (Stadler and Gökmen, 2024). This aspect is particularly critical in space-based food systems, where thermal processing under low gravity may enhance acrylamide formation due to uneven heat transfer and limited moisture dispersion (Kostoglou and Karapantsios, 2023). Minimizing asparagine accumulation under RB lighting could therefore represent an effective strategy to reduce process-induced contaminants and improve food safety in BLSSs.

In conclusion, potato plants grown in a prototype of multi-layer cultivation system with dynamic integration of RB LED light successfully completed the tuber-to-tuber cycle, producing healthy plants and tubers. Despite receiving a lower DLI, plants grown under supplemental RB LED light exhibited higher tuber yield than those grown under natural sunlight. However, this yield increase was accompanied by a reduced harvest index, suggesting greater aboveground biomass accumulation. Our integrated analysis indicated that plants modulated distinct metabolic responses depending on the lighting regime. Under supplemental LED lighting, physiological and metabolic reprogramming led to the accumulation of nitrogen, polyamines, and some of their precursor amino acids, supporting yield enhancement and improved stress tolerance. In contrast, plants exposed to only natural sunlight prioritized classical antioxidant and defensive strategies associated with augmented levels of stress-responsive proteins, polyphenols, ascorbic acid, and glutathione, in response to aging and photoinhibitory stress during the bulking stage. Notably, RB LED light under moderate DLI promoted the accumulation of health-beneficial metabolites such as polyamines, while simultaneously reducing undesirable glycoalkaloids and asparagine, thereby improving nutritional tuber quality. These findings underscore the role of light quality in modulating both nutritional and safety attributes of edible plant tissues and provide useful data for developing optimized lighting strategies for controlled-environment agriculture as well as for bioregenerative space farming systems.

## Data Availability

The datasets presented in this study can be found in online repositories. The names of the repository/repositories and accession number(s) can be found below: https://www.ncbi.nlm.nih.gov/, PRJNA1400930; https://www.ebi.ac.uk/pride/archive/; PXD07347, https://www.ebi.ac.uk/metabolights/, MetaboLights MTBLS13499.
