# Supplementary material for: Multi-omics analyses to assess the effects of multi-layer cultivation with supplemental LED lighting on potato yield and quality
Source: Front Plant Sci. 2026 Aug 19;17:1913161. doi: 10.3389/fpls.2026.1913161 (PMC13533967; doi:10.3389/fpls.2026.1913161)
Supplement: Supplementary file 1 [file DataSheet1.docx]

Supplementary Material

# 1 Supplemental Figures and legends

**Figure S1**. Leaf greenness, expressed as SPAD index, measured at different phenological stages in potato plants cv. ‘Colomba’ grown under natural light (CTRL) and LED-supplemented natural light (LED). Mean values ± standard error, n = 6. Different letters indicate significant differences according to Tukey’s multiple-range test at p < 0.05.

**Figure S2.** Total dry mass partitioning in the plant organs (percentage of total dry biomass) in potato cv. ‘Colomba’ grown under natural light (CTRL) and LED-supplemented natural light (LED).

**Figure S3.** Dot Plot of Gene Ontology (GO) and KEGG enrichment analyses of upregulated differentially expressed genes (DEGs) in S. tuberosum tubers under LED light treatment compared to control. The dot plots show the most significant enriched terms in (A) Biological Process (BP), (B) Cellular Component (CC), (C) Molecular Function (MF), and (D) KEGG pathways. Dot size indicates the number of associated DEGs, color represents the adjusted p-value (p.adjust), and the x-axis shows the gene ratio.

**Figure S4.** Dot Plot of Gene Ontology (GO) analysis of down-and over-represented proteins in *S. tuberosum* tubers of potato plants under LED light treatment compared to control. The dot plots show the most significant enriched terms in Biological Process (BP) in down- (A) and over-represented proteins (B). Dot size indicates the number of associated DRPs, colour represents the adjusted p-value (p.adjust), and the x-axis shows the protein ratio.

**Figure S5.** Primary and secondary metabolite variation in potato under CTRL and LED conditions. (A) 3D plot LC–MS/MS analysis in positive ion mode in the time range 0–25 min (x axis). The y axis reports the log2 fold change of the LED condition over the control counterpart; z axis summarizes the *m/z* value of each feature ranging from red (*m/z* 120) to purple (*m/z* 1500) representative of screened signals upon positive data analysis workﬂows over blank procedural samples. (B) Key metabolites annotated and identified in the tuber of potato grown in CTRL and LED conditions. The figure shows the response as area counts in LED and CTRL samples (n=4).

# 2 Supplemental Tables

**Table S1.** Daily Light Integral (DLI) recorded under natural light (CTRL) and LED-supplemented natural light (LED) at different phenological stages of potato plants. Mean values ± standard errors. Different letters indicate significant differences according to Tukey's multiple-range test (p<0.05). ** p<0.01; *** p<0.001.

|  |  | DLI (mol m^-2^ d^-1^) |
| --- | --- | --- |
| Lighting treatment (L) | CTRL | 10.37±0.99 a |
|  | LED | 8.24±0.45 b |
|  |  |  |
| Phenological stage (D) | Vegetative | 6.53±0.26 b |
|  | Flowering | 9.90±0.57 a |
|  | Tuber bulking | 11.48±1.15 a |
|  |  |  |
| LxD | Vegetative x CTRL | 6.32±0.27 c |
|  | Vegetative x LED | 6.73±0.47 c |
|  | Flowering x CTRL | 10.77±0.69 ab |
|  | Flowering x LED | 9.02±0.76 bc |
|  | Tuber bulking x CTRL | 14.02±1.52 a |
|  | Tuber bulking x LED | 8.95±0.66 bc |
|  |  |  |
| Significance | Treatment (L) | *** |
|  | Phenological stage (D) | ******* |
|  | L x D | ** |

**Table S2.** Tuber mineral profile (g kg⁻¹ or mg kg⁻¹ DW) of potato cv. ‘Colomba’ grown under natural light (CTRL) and LED-supplemented natural light (LED). Mean values ± standard errors; n = 6. ns, *, non-significant or significant at P ≤ 0.05, respectively, according to the Student's t-test.

|  | CTRL | LED | Significance |
| --- | --- | --- | --- |
| *(g kg^-1^ DW)* |  |  |  |
| K | 26.6 ± 0.2 | 25.4 ± 0.8 | ns |
| N | 9.8 ± 0.2 | 11.8 ± 0.6 | * |
| P | 4.1 ± 0.1 | 4.0 ± 0.2 | ns |
| S | 2.2 ± 0.1 | 2.0 ± 0.1 | ns |
| Mg | 1.3 ± 0.1 | 1.3 ± 0.1 | ns |
|  |  |  |  |
| *(mg kg^-1^ DW)* |  |  |  |
| Ca | 119.5 ± 10.7 | 106.8 ± 14.9 | ns |
| Na | 48.3 ± 2.0 | 49.6 ± 2.8 | ns |
| Fe | 27.2 ± 2.2 | 22.6 ± 1.2 | ns |
| Mn | 10.9 ± 1.1 | 10.8 ± 0.8 | ns |
| Zn | 8.1 ± 0.2 | 9.0 ± 0.6 | ns |
| B | 6.4 ± 0.1 | 5.8 ± 0.2 | * |
| Cu | 5.3 ± 0.2 | 4.9 ± 0.6 | ns |
| Mo | 1.6 ± 0.1 | 1.5 ± 0.1 | ns |

**Table S3.** Functional categories of differentially expressed genes grouped by biological process.

| Process/pathway | Regulation | Gene annotation |
| --- | --- | --- |
| Carotenoids | Upregulated | Abscisic-Aldehyde Oxidase (AAO3) |
| Carotenoids | Upregulated | Carotene Epsilon-Monooxygenase/LUT1 |
| Carotenoids | Upregulated | Phytoene Synthase (crtB) |
| Sucrose | Upregulated | Glycosyltransferase/Sucrose-Phosphate Synthase 1-Related |
| Sucrose | Upregulated | Sucrose Synthase (E2.4.1.13) |
| Sucrose | Upregulated | Sucrose-Phosphate Synthase 3-Related |
| Glucose | Upregulated | Glyceraldehyde-3-Phosphate Dehydrogenase Gapa1, Chloroplastic-Related |
| Glucose | Upregulated | Glucose-6-Phosphate 1-Dehydrogenase 1, Chloroplastic |
| Glucose/trehalose | Upregulated | Alpha,Alpha-Trehalose-Phosphate Synthase (Udp-Forming) 11-Related |
| AA | Upregulated | L-3-cyanoalanine synthase / cysteine synthase (ATCYSC1) |
| AA | Upregulated | Cystathionine gamma-synthase / O-succinylhomoserine synthetase |
| AA | Upregulated | Proline Transporter 1-Related |
| AA | Upregulated | S-Adenosylmethionine Synthase 4 |
| Polyphenols | Upregulated | Chalcone Synthase (CHS) |
| Polyphenols | Upregulated | Chalcone-Flavonone Isomerase 3-Related |
| Hormones | Upregulated | Adenylate Isopentenyltransferase (Cytokinin Synthase) (IPT) |
| Glycoalkaloids | Downregulated | Sterol 24-C-Methyltransferase (E2.1.1.41, SMT1, ERG6) |
| Glycoalkaloids | Upregulated | Cytochrome P450 Family Member/ subfamily not named |
| Stress-related | Downregulated | Small Heat-Shock Protein HSP20 Family/ subfamily not named |
| Stress-related | Downregulated | Heat Shock Protein 90-1 |
| Stress-related | Downregulated | Universal stress protein family (USP) |
| Stress-related | Downregulated | Small Heat-Shock Protein Hsp20 Family/ subfamily not named |
| Stress-related | Downregulated | 15.7 Kda Heat Shock Protein, Peroxisomal |
| Stress-related | Downregulated | Small Heat-Shock Protein Hsp20 Family/17.6 Kda Class I Heat Shock Protein 1-Related |

**Table S4.** GO enrichment analysis of up-regulated genes (CTRL vs LED), showing significantly over-represented GO biological process terms (FDR-adjusted; see Methods), mainly related to photosynthesis, light harvesting, chlorophyll biosynthesis, and redox homeostasis.

**Table S5.**  KEGG enrichment analysis of up-regulated genes (CTRL vs LED), reporting significantly over-represented KEGG pathways (FDR-adjusted; see Methods), including carbon fixation, and pigment metabolism.

**Table S6.** List of identified and differentially expressed protein species detected between LED-treated and CTRL potato samples. Differentially abundant proteins were defined according to the following thresholds: up‑regulated proteins with _ Fold Change (FC) > 1.2_; down‑regulated proteins with _FC < 0.6_; and _ p‑value < 0.05_. Proteins were identified and quantified by nanoESI‑LC–MS/MS analysis of _Solanum tuberosum_ samples. The raw data were processed with MaxQuant (version MaxQuant_v2.7.5.0) using the Andromeda search engine. Spectra were searched against the UniProt Solanum tuberosum reference proteome, including common contaminants. Label‑free quantification (LFQ) was performed using MaxQuant default parameters unless otherwise specified. Subsequent statistical analysis, including filtering and differential abundance testing, was carried out using Perseus (version _v2.1.5.0).

| **Protein IDs** | **Gene Name** | **Description** | **Peptides** | **Sequence coverage [%]** | **Mol. weight [kDa]** | **Score** | **sample (CTRL) Intensity** | **sample (LED) Intensity** | **Fold Change** | **Regulation** | **C: Student's T-test Significant CTRL_LED** | **N: -Log Student's T-test p-value CTRL_LED** | **Pvalue** | **N: Student's T-test Difference CTRL_LED** | **N: Student's T-test Test statistic CTRL_LED** |
| --- | --- | --- | --- | --- | --- | --- | --- | --- | --- | --- | --- | --- | --- | --- | --- |
| M1D193 | 102578911 | tr\|M1D193\|M1D193_SOLTU procollagen-proline 4-dioxygenase OS=Solanum tuberosum OX=4113 GN=102578911 PE=3 SV=1 | 2 | 8,5 | 32,739 | 43,494 | 2,24E+07 | 6,48E+06 | 2,89E-01 | DOWN | + | 1,47735 | 0,033 | 14,8864 | 2,56635 |
| M1C0N9 | 102606407 | tr\|M1C0N9\|M1C0N9_SOLTU Sulfurtransferase OS=Solanum tuberosum OX=4113 GN=102606407 PE=4 SV=1 | 3 | 12,9 | 41,048 | 32,702 | 3,29E+07 | 2,11E+07 | 6,41E-01 | DOWN | + | 2,64541 | 0,002 | 0,663026 | 4,40798 |
| M0ZU86 | 102583725 | tr\|M0ZU86\|M0ZU86_SOLTU Nucleic acid binding protein OS=Solanum tuberosum OX=4113 GN=102583725 PE=4 SV=1 | 3 | 16,2 | 29,496 | 20,074 | 1,59E+07 | 4,49E+06 | 2,82E-01 | DOWN | + | 1,49387 | 0,032 | 14,6814 | 2,59081 |
| M1B164 | 102602739 | tr\|M1B164\|M1B164_SOLTU Acyl-protein thioesterase 1,2 OS=Solanum tuberosum OX=4113 GN=102602739 PE=3 SV=1 | 3 | 17,7 | 27,878 | 47,972 | 1,55E+07 | 2,64E+06 | 1,70E-01 | DOWN | + | 1,58259 | 0,026 | 14,9221 | 2,72253 |
| M1D4K8 | 102580232 | tr\|M1D4K8\|M1D4K8_SOLTU Polygalacturonase OS=Solanum tuberosum OX=4113 PE=3 SV=1 | 3 | 10,9 | 49,774 | 21,08 | 1,40E+07 | 2,15E+06 | 1,54E-01 | DOWN | + | 1,6064 | 0,025 | 14,9144 | 2,75799 |
| M1A3E1 | 102581154 | tr\|M1A3E1\|M1A3E1_SOLTU DOMON domain-containing protein OS=Solanum tuberosum OX=4113 GN=102581154 PE=4 SV=1 | 4 | 20,5 | 26,004 | 308,31 | 1,04E+08 | 8,21E+07 | 7,89E-01 | DOWN | + | 1,45713 | 0,035 | 0,340221 | 2,53644 |
| M1A1V7 | PPA1 | tr\|M1A1V7\|M1A1V7_SOLTU inorganic diphosphatase OS=Solanum tuberosum OX=4113 GN=102584131 PE=3 SV=1;sp\|Q43187\|IPYR_SOLTU Soluble inorganic pyrophosphatase PPA1 OS=Solanum tuberosum OX=4113 GN=PPA1 PE=1 SV=1 | 4 | 39,3 | 24,234 | 132,57 | 1,65E+08 | 1,21E+08 | 7,33E-01 | DOWN | + | 2,23816 | 0,006 | 0,445778 | 3,73107 |
| M1BHW0 | 102590570 | tr\|M1BHW0\|M1BHW0_SOLTU NADH-ubiquinone oxidoreductase 11 kDa subunit OS=Solanum tuberosum OX=4113 GN=102590570 PE=4 SV=1 | 4 | 70,1 | 10,754 | 32,086 | 4,71E+07 | 3,15E+07 | 6,69E-01 | DOWN | + | 1,86852 | 0,014 | 0,589999 | 3,15307 |
| M0ZVI3 | 102585875 | tr\|M0ZVI3\|M0ZVI3_SOLTU inorganic diphosphatase OS=Solanum tuberosum OX=4113 GN=102585875 PE=3 SV=1;tr\|M0ZVI2\|M0ZVI2_SOLTU inorganic diphosphatase OS=Solanum tuberosum OX=4113 GN=102585875 PE=3 SV=1 | 4 | 42,8 | 21,746 | 30,376 | 6,39E+07 | 4,20E+07 | 6,57E-01 | DOWN | + | 3,0774 | 0,001 | 0,602172 | 5,18583 |
| M1C6Y7 | 102585385 | tr\|M1C6Y7\|M1C6Y7_SOLTU Acidic ribosomal protein P1a OS=Solanum tuberosum OX=4113 GN=102585385 PE=3 SV=1 | 4 | 78,6 | 11,319 | 96,02 | 1,06E+09 | 6,88E+08 | 6,49E-01 | DOWN | + | 1,3962 | 0,040 | 0,709091 | 2,44641 |
| M1CEB0 | 102579804 | tr\|M1CEB0\|M1CEB0_SOLTU Uncharacterized protein OS=Solanum tuberosum OX=4113 GN=102579804 PE=3 SV=1 | 4 | 34,3 | 18,615 | 57,065 | 9,14E+07 | 5,72E+07 | 6,26E-01 | DOWN | + | 1,7009 | 0,020 | 0,68355 | 2,89936 |
| M1A0Z7 | 102598867 | tr\|M1A0Z7\|M1A0Z7_SOLTU Mitochondrial small heat shock protein OS=Solanum tuberosum OX=4113 GN=102598867 PE=3 SV=1 | 4 | 28,9 | 23,912 | 323,31 | 4,27E+08 | 1,82E+08 | 4,26E-01 | DOWN | + | 4,76626 | 0,000 | 1,22601 | 9,09673 |
| M1AJ64 | 102577794 | tr\|M1AJ64\|M1AJ64_SOLTU Small heat-shock protein homolog protein OS=Solanum tuberosum OX=4113 GN=102603518 PE=3 SV=1 | 4 | 25,9 | 22,355 | 85,823 | 4,50E+08 | 1,35E+08 | 3,00E-01 | DOWN | + | 5,18828 | 0,000 | 1,72885 | 10,3669 |
| M1ATE5 | 102599235 | tr\|M1ATE5\|M1ATE5_SOLTU Hsp90 co-chaperone AHA1 OS=Solanum tuberosum OX=4113 GN=102599235 PE=3 SV=1 | 5 | 20,2 | 39,326 | 62,572 | 2,19E+07 | 1,76E+07 | 8,04E-01 | DOWN | + | 1,49916 | 0,032 | 0,312356 | 2,59865 |
| M1CVD2 | 102588644 | tr\|M1CVD2\|M1CVD2_SOLTU tryptophan synthase OS=Solanum tuberosum OX=4113 GN=102588644 PE=3 SV=1;tr\|M1CVD1\|M1CVD1_SOLTU tryptophan synthase OS=Solanum tuberosum OX=4113 GN=102588644 PE=4 SV=1 | 5 | 17,5 | 54,129 | 55,771 | 2,50E+07 | 1,98E+07 | 7,92E-01 | DOWN | + | 1,45371 | 0,035 | 0,342847 | 2,53138 |
| M1D3Q7 | 102579186 | tr\|M1D3Q7\|M1D3Q7_SOLTU Ethylene response protein OS=Solanum tuberosum OX=4113 GN=102579186 PE=4 SV=1 | 5 | 40 | 19,576 | 34,406 | 3,72E+07 | 2,94E+07 | 7,90E-01 | DOWN | + | 2,5121 | 0,003 | 0,336493 | 4,181 |
| M1C6J2 | 102597556 | tr\|M1C6J2\|M1C6J2_SOLTU Ubiquitin-protein ligase OS=Solanum tuberosum OX=4113 GN=102597556 PE=4 SV=1 | 5 | 14,5 | 45,824 | 89,26 | 4,27E+07 | 3,07E+07 | 7,19E-01 | DOWN | + | 1,9636 | 0,011 | 0,455791 | 3,29906 |
| M1BYS1 | 102601370 | tr\|M1BYS1\|M1BYS1_SOLTU Furin OS=Solanum tuberosum OX=4113 GN=102601370 PE=3 SV=1 | 5 | 32,9 | 32,316 | 323,31 | 1,16E+08 | 7,84E+07 | 6,76E-01 | DOWN | + | 3,29367 | 0,001 | 0,576543 | 5,60287 |
| M0ZSL5 | 102602804 | tr\|M0ZSL5\|M0ZSL5_SOLTU Dihydrolipoyl dehydrogenase OS=Solanum tuberosum OX=4113 GN=102602804 PE=3 SV=1 | 5 | 12,1 | 60,253 | 109,43 | 4,01E+07 | 2,62E+07 | 6,53E-01 | DOWN | + | 2,72729 | 0,002 | 0,616631 | 4,55026 |
| M1CZP5 | 102589666 | tr\|M1CZP5\|M1CZP5_SOLTU Peptidyl-prolyl cis-trans isomerase OS=Solanum tuberosum OX=4113 GN=102589666 PE=3 SV=1 | 5 | 30 | 22,181 | 323,31 | 2,27E+08 | 1,37E+08 | 6,04E-01 | DOWN | + | 1,7411 | 0,018 | 0,702954 | 2,95983 |
| K7VKA6 | Sl20.1shsp | tr\|K7VKA6\|K7VKA6_SOLTU 17.6 kD class I small heat shock protein OS=Solanum tuberosum OX=4113 GN=Sl20.1shsp PE=2 SV=1 | 5 | 43,5 | 17,652 | 323,31 | 3,53E+09 | 1,40E+09 | 3,97E-01 | DOWN | + | 4,423 | 0,000 | 1,30634 | 8,16297 |
| M1CZL5 | 102605425 | tr\|M1CZL5\|M1CZL5_SOLTU 17.6 kD class I small heat shock protein OS=Solanum tuberosum OX=4113 GN=102605425 PE=3 SV=1 | 5 | 43,5 | 17,635 | 323,31 | 3,54E+08 | 9,90E+07 | 2,80E-01 | DOWN | + | 3,57204 | 0,000 | 1,75021 | 6,17059 |
| M1BLV1 | 102598915 | tr\|M1BLV1\|M1BLV1_SOLTU Thioredoxin reductase OS=Solanum tuberosum OX=4113 GN=102598915 PE=3 SV=1 | 5 | 22,3 | 34,871 | 65,071 | 1,72E+07 | 3,75E+06 | 2,18E-01 | DOWN | + | 1,53858 | 0,029 | 14,89 | 2,65711 |
| M1AT83 | 102592958 | tr\|M1AT83\|M1AT83_SOLTU Pre-mRNA-splicing factor SF2 OS=Solanum tuberosum OX=4113 GN=102592958 PE=4 SV=1;tr\|M1AT81\|M1AT81_SOLTU Pre-mRNA-splicing factor SF2 OS=Solanum tuberosum OX=4113 GN=102592958 PE=4 SV=1 | 5 | 41,5 | 17,3 | 20,246 | 3,28E+07 | 6,63E+06 | 2,02E-01 | DOWN | + | 1,53208 | 0,029 | 15,3846 | 2,64747 |
| M1CSR8 | 102605090 | tr\|M1CSR8\|M1CSR8_SOLTU Peptidyl-prolyl cis-trans isomerase OS=Solanum tuberosum OX=4113 GN=102605090 PE=3 SV=1 | 5 | 36 | 18,847 | 323,31 | 1,17E+08 | 2,19E+07 | 1,87E-01 | DOWN | + | 1,54789 | 0,028 | 16,631 | 2,67094 |
| M1CVE4 | 102596065 | tr\|M1CVE4\|M1CVE4_SOLTU Serine hydroxymethyltransferase OS=Solanum tuberosum OX=4113 GN=102596065 PE=3 SV=1;tr\|M1CVE3\|M1CVE3_SOLTU Serine hydroxymethyltransferase OS=Solanum tuberosum OX=4113 GN=102596065 PE=3 SV=1 | 6 | 16,3 | 56,964 | 67,571 | 4,35E+07 | 3,51E+07 | 8,07E-01 | DOWN | + | 1,61325 | 0,024 | 0,30154 | 2,7682 |
| M0ZVJ6 | 102591288 | tr\|M0ZVJ6\|M0ZVJ6_SOLTU Proteasome subunit beta OS=Solanum tuberosum OX=4113 GN=102591288 PE=3 SV=1 | 6 | 39,9 | 24,618 | 131,75 | 1,23E+08 | 9,89E+07 | 8,04E-01 | DOWN | + | 1,64048 | 0,023 | 0,30039 | 2,80885 |
| M1A383 | 102606145 | tr\|M1A383\|M1A383_SOLTU Calreticulin OS=Solanum tuberosum OX=4113 GN=102606145 PE=3 SV=1;tr\|M1A384\|M1A384_SOLTU Calreticulin OS=Solanum tuberosum OX=4113 GN=102606145 PE=3 SV=1 | 6 | 19,5 | 48,252 | 36,95 | 2,12E+07 | 1,67E+07 | 7,88E-01 | DOWN | + | 1,62539 | 0,024 | 0,330931 | 2,78632 |
| M1D6Y3 | 102589601 | tr\|M1D6Y3\|M1D6Y3_SOLTU 60S ribosomal protein L12 OS=Solanum tuberosum OX=4113 GN=102589601 PE=3 SV=1;tr\|K7WTX4\|K7WTX4_SOLTU 60S ribosomal protein L12 OS=Solanum tuberosum OX=4113 GN=102600463 PE=2 SV=1 | 6 | 50,6 | 17,784 | 239,04 | 7,81E+08 | 6,13E+08 | 7,85E-01 | DOWN | + | 1,59696 | 0,025 | 0,339873 | 2,74393 |
| M1B9J5 | 102587356 | tr\|M1B9J5\|M1B9J5_SOLTU Nutrient reservoir OS=Solanum tuberosum OX=4113 GN=102587356 PE=3 SV=1;tr\|M1B9J6\|M1B9J6_SOLTU Nutrient reservoir OS=Solanum tuberosum OX=4113 GN=102587356 PE=3 SV=1 | 6 | 25,6 | 38,206 | 64,995 | 4,59E+07 | 3,40E+07 | 7,41E-01 | DOWN | + | 1,98584 | 0,010 | 0,419715 | 3,33344 |
| M1AEB9 | 102604796 | tr\|M1AEB9\|M1AEB9_SOLTU Beta-galactosidase OS=Solanum tuberosum OX=4113 GN=102604796 PE=3 SV=1;tr\|M1AEC1\|M1AEC1_SOLTU Beta-galactosidase OS=Solanum tuberosum OX=4113 GN=102604796 PE=3 SV=1;tr\|M1AEC0\|M1AEC0_SOLTU Beta-galactosidase OS=Solanum tuberosum OX=41 | 6 | 11 | 93,091 | 161,01 | 1,92E+07 | 1,37E+07 | 7,14E-01 | DOWN | + | 1,69389 | 0,020 | 0,479401 | 2,88883 |
| M1C015 | 102580720 | tr\|M1C015\|M1C015_SOLTU hydroxymethylbilane synthase OS=Solanum tuberosum OX=4113 GN=102580720 PE=3 SV=1 | 6 | 22,5 | 39,176 | 81,464 | 8,02E+07 | 5,64E+07 | 7,03E-01 | DOWN | + | 2,51094 | 0,003 | 0,494532 | 4,17904 |
| M1BYS8 | 102604529 | tr\|M1BYS8\|M1BYS8_SOLTU Class II small heat shock protein Le-HSP17.6 OS=Solanum tuberosum OX=4113 GN=102604529 PE=3 SV=1;tr\|M1AEP4\|M1AEP4_SOLTU Class II small heat shock protein Le-HSP17.6 OS=Solanum tuberosum OX=4113 GN=102602952 PE=3 SV=1 | 6 | 60,1 | 17,686 | 323,31 | 1,36E+09 | 4,04E+08 | 2,97E-01 | DOWN | + | 5,29361 | 0,000 | 1,73111 | 10,7069 |
| M1AEK1 | 102598516 | tr\|M1AEK1\|M1AEK1_SOLTU Inosine-5-monophosphate dehydrogenase OS=Solanum tuberosum OX=4113 GN=102598516 PE=3 SV=1 | 6 | 20,5 | 52,827 | 47,683 | 3,13E+07 | 9,06E+06 | 2,89E-01 | DOWN | + | 1,46715 | 0,034 | 15,1127 | 2,55126 |
| M1BTS7 | 102600485 | tr\|M1BTS7\|M1BTS7_SOLTU 177 protein OS=Solanum tuberosum OX=4113 GN=102600485 PE=3 SV=1 | 7 | 60,7 | 19,449 | 304,9 | 4,72E+08 | 3,70E+08 | 7,84E-01 | DOWN | + | 1,86652 | 0,014 | 0,343655 | 3,15002 |
| M1CWA6 | 102578706 | tr\|M1CWA6\|M1CWA6_SOLTU acetyl-CoA C-acyltransferase OS=Solanum tuberosum OX=4113 GN=102578706 PE=3 SV=1 | 7 | 27,3 | 48,938 | 51,605 | 4,12E+07 | 3,18E+07 | 7,72E-01 | DOWN | + | 1,49453 | 0,032 | 0,373082 | 2,59179 |
| M1CYK7 | 102595083 | tr\|M1CYK7\|M1CYK7_SOLTU Stem-specific protein TSJT1 OS=Solanum tuberosum OX=4113 GN=102595083 PE=4 SV=1 | 7 | 35,1 | 27,198 | 75,732 | 2,24E+08 | 1,67E+08 | 7,46E-01 | DOWN | + | 2,29749 | 0,005 | 0,423198 | 3,82678 |
| M1D5J8 | 102595383 | tr\|M1D5J8\|M1D5J8_SOLTU Homocysteine s-methyltransferase OS=Solanum tuberosum OX=4113 GN=102595383 PE=4 SV=1 | 7 | 27,2 | 36,599 | 69,881 | 1,27E+08 | 9,32E+07 | 7,34E-01 | DOWN | + | 2,29766 | 0,005 | 0,431483 | 3,82706 |
| M1AE74 | 102577546 | tr\|M1AE74\|M1AE74_SOLTU Lactoylglutathione lyase OS=Solanum tuberosum OX=4113 GN=102577546 PE=3 SV=1;tr\|M1AE75\|M1AE75_SOLTU Lactoylglutathione lyase OS=Solanum tuberosum OX=4113 GN=102577546 PE=3 SV=1 | 7 | 54,6 | 20,781 | 54,516 | 1,49E+08 | 1,02E+08 | 6,85E-01 | DOWN | + | 1,59994 | 0,025 | 0,548756 | 2,74837 |
| Q9XF12 | 102586427 | tr\|Q9XF12\|Q9XF12_SOLTU Peptidyl-prolyl cis-trans isomerase OS=Solanum tuberosum OX=4113 GN=102586427 PE=2 SV=1 | 7 | 67,3 | 17,925 | 323,31 | 2,56E+09 | 1,75E+09 | 6,84E-01 | DOWN | + | 1,70109 | 0,020 | 0,543174 | 2,89965 |
| M0ZJ08 | 102601245 | tr\|M0ZJ08\|M0ZJ08_SOLTU Agmatine deiminase OS=Solanum tuberosum OX=4113 GN=102601245 PE=3 SV=1 | 7 | 26,1 | 41,658 | 77,064 | 1,55E+08 | 9,94E+07 | 6,41E-01 | DOWN | + | 2,47407 | 0,003 | 0,645808 | 4,11727 |
| M1CMY9 | 102577566 | tr\|M1CMY9\|M1CMY9_SOLTU Superoxide dismutase OS=Solanum tuberosum OX=4113 GN=102588174 PE=3 SV=1;tr\|M1CMZ4\|M1CMZ4_SOLTU Superoxide dismutase OS=Solanum tuberosum OX=4113 GN=102588174 PE=3 SV=1;tr\|M1CMZ3\|M1CMZ3_SOLTU Superoxide dismutase OS=Solanum tuberosum | 7 | 43,3 | 28,323 | 136,23 | 2,49E+08 | 1,51E+08 | 6,06E-01 | DOWN | + | 3,91344 | 0,000 | 0,71738 | 6,91989 |
| M1CSH0 | 102578969 | tr\|M1CSH0\|M1CSH0_SOLTU Small heat-shock protein OS=Solanum tuberosum OX=4113 GN=102578969 PE=3 SV=1 | 7 | 45 | 26,191 | 323,31 | 5,78E+08 | 1,24E+08 | 2,15E-01 | DOWN | + | 5,04031 | 0,000 | 2,19395 | 9,90517 |
| M1C340 | 102596301 | tr\|M1C340\|M1C340_SOLTU aspartate-semialdehyde dehydrogenase OS=Solanum tuberosum OX=4113 GN=102596301 PE=3 SV=1 | 8 | 30,2 | 42,011 | 108,33 | 1,07E+08 | 9,18E+07 | 8,58E-01 | DOWN | + | 1,39691 | 0,040 | 0,223681 | 2,44747 |
| M1CC20 | 102591839 | tr\|M1CC20\|M1CC20_SOLTU Formamidase OS=Solanum tuberosum OX=4113 GN=102591839 PE=4 SV=1 | 8 | 25,1 | 50,954 | 206,68 | 8,04E+07 | 6,47E+07 | 8,05E-01 | DOWN | + | 1,39719 | 0,040 | 0,30503 | 2,44788 |
| M1A4U5 | 102593955 | tr\|M1A4U5\|M1A4U5_SOLTU Aldose 1-epimerase OS=Solanum tuberosum OX=4113 GN=102593955 PE=3 SV=1;tr\|M1A4U4\|M1A4U4_SOLTU Aldose-1-epimerase OS=Solanum tuberosum OX=4113 GN=102593955 PE=3 SV=1 | 8 | 38,5 | 36,574 | 61,864 | 5,16E+07 | 3,65E+07 | 7,07E-01 | DOWN | + | 1,35996 | 0,044 | 0,473697 | 2,39293 |
| M1A2T5 | 102602349 | tr\|M1A2T5\|M1A2T5_SOLTU Peptidyl-prolyl cis-trans isomerase OS=Solanum tuberosum OX=4113 GN=102602349 PE=3 SV=1;tr\|M1A2T6\|M1A2T6_SOLTU Peptidyl-prolyl cis-trans isomerase OS=Solanum tuberosum OX=4113 GN=102602349 PE=3 SV=1 | 8 | 46 | 26,526 | 166,64 | 2,20E+08 | 1,45E+08 | 6,59E-01 | DOWN | + | 1,91056 | 0,012 | 0,598215 | 3,21742 |
| M0ZWB1 | 102597275 | tr\|M0ZWB1\|M0ZWB1_SOLTU Protein yrdA OS=Solanum tuberosum OX=4113 GN=102597275 PE=3 SV=1 | 9 | 50,2 | 29,523 | 229,97 | 8,36E+07 | 6,45E+07 | 7,72E-01 | DOWN | + | 1,79848 | 0,016 | 0,367375 | 3,04653 |
| M1AKV3 | 102599627 | tr\|M1AKV3\|M1AKV3_SOLTU Proteasome subunit alpha type OS=Solanum tuberosum OX=4113 GN=102599627 PE=3 SV=1;tr\|M1AKV2\|M1AKV2_SOLTU Proteasome subunit alpha type-5 OS=Solanum tuberosum OX=4113 GN=102599627 PE=3 SV=1 | 9 | 57,4 | 26,068 | 323,31 | 1,87E+08 | 1,42E+08 | 7,59E-01 | DOWN | + | 1,81306 | 0,015 | 0,400284 | 3,06866 |
| Q38M52 | 102577817 | tr\|Q38M52\|Q38M52_SOLTU Proteasome subunit alpha type OS=Solanum tuberosum OX=4113 GN=102577817 PE=2 SV=1;tr\|M1API7\|M1API7_SOLTU Proteasome subunit alpha type OS=Solanum tuberosum OX=4113 GN=102577817 PE=3 SV=1 | 9 | 55,3 | 25,665 | 264,28 | 3,67E+08 | 2,76E+08 | 7,52E-01 | DOWN | + | 1,84476 | 0,014 | 0,41738 | 3,11684 |
| M1BU57 | 102599893 | tr\|M1BU57\|M1BU57_SOLTU EBP1 OS=Solanum tuberosum OX=4113 GN=102599893 PE=3 SV=1;tr\|M1BU58\|M1BU58_SOLTU EBP1 OS=Solanum tuberosum OX=4113 GN=102599893 PE=3 SV=1 | 9 | 24,1 | 42,908 | 61,077 | 2,65E+07 | 1,89E+07 | 7,13E-01 | DOWN | + | 1,97444 | 0,011 | 0,519398 | 3,31579 |
| M1DJ69 | 102578188 | tr\|M1DJ69\|M1DJ69_SOLTU Low molecular weight heat-shock protein OS=Solanum tuberosum OX=4113 GN=102578188 PE=3 SV=1 | 9 | 66 | 17,336 | 266,54 | 9,25E+09 | 5,37E+09 | 5,81E-01 | DOWN | + | 3,00556 | 0,001 | 0,757646 | 5,05161 |
| M1D249 | 102604604 | tr\|M1D249\|M1D249_SOLTU Alcohol dehydrogenase OS=Solanum tuberosum OX=4113 GN=102604604 PE=3 SV=1 | 10 | 37,1 | 41,032 | 79,801 | 7,07E+07 | 6,05E+07 | 8,56E-01 | DOWN | + | 1,50249 | 0,031 | 0,219492 | 2,60359 |
| M1CFZ0 | 102583061 | tr\|M1CFZ0\|M1CFZ0_SOLTU N-acetyl-gamma-glutamyl-phosphate reductase OS=Solanum tuberosum OX=4113 GN=102583061 PE=3 SV=1;tr\|M1CFY8\|M1CFY8_SOLTU N-acetyl-gamma-glutamyl-phosphate reductase, chloroplastic OS=Solanum tuberosum OX=4113 PE=3 SV=1 | 10 | 42,7 | 44,181 | 71,784 | 7,41E+07 | 5,17E+07 | 6,98E-01 | DOWN | + | 2,32186 | 0,005 | 0,526002 | 3,86636 |
| Q2VEH0 | atpB | sp\|Q2VEH0\|ATPB_SOLTU ATP synthase subunit beta, chloroplastic OS=Solanum tuberosum OX=4113 GN=atpB PE=3 SV=1;tr\|M0ZKF0\|M0ZKF0_SOLTU H(+)-transporting two-sector ATPase OS=Solanum tuberosum OX=4113 PE=3 SV=1 | 10 | 29,9 | 53,508 | 92,507 | 4,05E+07 | 2,81E+07 | 6,94E-01 | DOWN | + | 3,46291 | 0,000 | 0,52665 | 5,94364 |
| M1CTZ5 | 102603227 | tr\|M1CTZ5\|M1CTZ5_SOLTU Beta-ureidopropionase OS=Solanum tuberosum OX=4113 GN=102603227 PE=4 SV=1;tr\|M1CTZ6\|M1CTZ6_SOLTU Beta-ureidopropionase OS=Solanum tuberosum OX=4113 GN=102603227 PE=4 SV=1;tr\|M1CTZ4\|M1CTZ4_SOLTU Beta-ureidopropionase OS=Solanum tubero | 10 | 32,9 | 45,782 | 235,94 | 2,36E+08 | 1,59E+08 | 6,74E-01 | DOWN | + | 2,47275 | 0,003 | 0,569257 | 4,11506 |
| M1CXI3 | 102605051 | tr\|M1CXI3\|M1CXI3_SOLTU Importin subunit alpha OS=Solanum tuberosum OX=4113 GN=102605051 PE=3 SV=1 | 11 | 31,9 | 58,401 | 214,51 | 1,04E+08 | 8,90E+07 | 8,56E-01 | DOWN | + | 1,33127 | 0,047 | 0,212931 | 2,35062 |
| M1C911 | 102592332 | tr\|M1C911\|M1C911_SOLTU Peroxidase OS=Solanum tuberosum OX=4113 GN=102592332 PE=3 SV=1 | 11 | 55,6 | 35,077 | 287,05 | 2,31E+08 | 1,93E+08 | 8,35E-01 | DOWN | + | 1,48144 | 0,033 | 0,250492 | 2,57242 |
| M0ZMF8 | 102591400 | tr\|M0ZMF8\|M0ZMF8_SOLTU Phosphoserine aminotransferase OS=Solanum tuberosum OX=4113 GN=102591400 PE=3 SV=1 | 11 | 32,5 | 47,766 | 163,85 | 7,07E+07 | 5,57E+07 | 7,88E-01 | DOWN | + | 1,65693 | 0,022 | 0,336834 | 2,83345 |
| M1BJQ9 | 102585459 | tr\|M1BJQ9\|M1BJQ9_SOLTU Elongation factor Tu OS=Solanum tuberosum OX=4113 GN=102585459 PE=3 SV=1 | 11 | 38,4 | 51,882 | 243,46 | 6,64E+07 | 3,50E+07 | 5,27E-01 | DOWN | + | 3,39562 | 0,000 | 0,913394 | 5,80657 |
| M0ZNB0 | 102578720 | tr\|M0ZNB0\|M0ZNB0_SOLTU Ascorbate peroxidase OS=Solanum tuberosum OX=4113 GN=102578720 PE=3 SV=1 | 12 | 68,8 | 27,585 | 323,31 | 1,40E+09 | 1,05E+09 | 7,50E-01 | DOWN | + | 1,73143 | 0,019 | 0,39702 | 2,94527 |
| M0ZJ17 | 102581673 | tr\|M0ZJ17\|M0ZJ17_SOLTU Protein disulfide isomerase family OS=Solanum tuberosum OX=4113 GN=102581673 PE=3 SV=1 | 12 | 36,3 | 47,16 | 274,17 | 3,48E+08 | 2,58E+08 | 7,41E-01 | DOWN | + | 1,65612 | 0,022 | 0,413455 | 2,83224 |
| M0ZWY7 | 102602312 | tr\|M0ZWY7\|M0ZWY7_SOLTU Elongation factor Tu OS=Solanum tuberosum OX=4113 GN=102602312 PE=3 SV=1 | 12 | 38,7 | 49,061 | 323,31 | 1,56E+08 | 1,15E+08 | 7,37E-01 | DOWN | + | 2,2337 | 0,006 | 0,438453 | 3,72391 |
| M1C547 | 102595093 | tr\|M1C547\|M1C547_SOLTU isopentenyl-diphosphate Delta-isomerase OS=Solanum tuberosum OX=4113 GN=102595093 PE=3 SV=1;tr\|M1AB35\|M1AB35_SOLTU isopentenyl-diphosphate Delta-isomerase OS=Solanum tuberosum OX=4113 GN=102580973 PE=3 SV=1 | 13 | 66,8 | 27,168 | 136,06 | 4,13E+08 | 3,62E+08 | 8,76E-01 | DOWN | + | 1,38611 | 0,041 | 0,189114 | 2,43152 |
| M1D5Y7 | 102593524 | tr\|M1D5Y7\|M1D5Y7_SOLTU glutamate-1-semialdehyde 2,1-aminomutase OS=Solanum tuberosum OX=4113 GN=102593524 PE=3 SV=1;tr\|M1D5Y8\|M1D5Y8_SOLTU glutamate-1-semialdehyde 2,1-aminomutase OS=Solanum tuberosum OX=4113 GN=102593524 PE=3 SV=1 | 13 | 42,2 | 51,433 | 323,31 | 2,17E+08 | 1,85E+08 | 8,53E-01 | DOWN | + | 1,5075 | 0,031 | 0,227858 | 2,61101 |
| M1AVG8 | 102587813 | tr\|M1AVG8\|M1AVG8_SOLTU CPN60A OS=Solanum tuberosum OX=4113 GN=102587813 PE=3 SV=1 | 13 | 33,3 | 62,469 | 191,73 | 7,40E+07 | 5,99E+07 | 8,09E-01 | DOWN | + | 2,59588 | 0,003 | 0,299734 | 4,32299 |
| M1C6L7 | PURA | tr\|M1C6L7\|M1C6L7_SOLTU Adenylosuccinate synthetase, chloroplastic OS=Solanum tuberosum OX=4113 GN=PURA PE=3 SV=1 | 13 | 36,5 | 54,75 | 225,83 | 1,49E+08 | 1,20E+08 | 8,05E-01 | DOWN | + | 2,37064 | 0,004 | 0,311183 | 3,94606 |
| M1BS59 | 102604637 | tr\|M1BS59\|M1BS59_SOLTU 26S proteasome subunit 4 OS=Solanum tuberosum OX=4113 GN=102604637 PE=3 SV=1;tr\|M1BAK6\|M1BAK6_SOLTU 26S proteasome subunit 4 OS=Solanum tuberosum OX=4113 PE=3 SV=1 | 14 | 40,8 | 49,435 | 323,31 | 2,11E+08 | 1,68E+08 | 7,96E-01 | DOWN | + | 2,08079 | 0,008 | 0,329761 | 3,48141 |
| M1BCZ9 | 102599485 | tr\|M1BCZ9\|M1BCZ9_SOLTU Lactoylglutathione lyase OS=Solanum tuberosum OX=4113 GN=102599485 PE=3 SV=1 | 14 | 58,2 | 32,811 | 323,31 | 2,05E+09 | 1,52E+09 | 7,41E-01 | DOWN | + | 3,14456 | 0,001 | 0,430456 | 5,31321 |
| M1BPZ5 | 102598930 | tr\|M1BPZ5\|M1BPZ5_SOLTU malate dehydrogenase OS=Solanum tuberosum OX=4113 GN=102598930 PE=3 SV=1 | 14 | 56,1 | 43,275 | 323,31 | 8,87E+08 | 5,92E+08 | 6,67E-01 | DOWN | + | 2,1669 | 0,007 | 0,568925 | 3,61729 |
| M1CAU1 | 102580368 | tr\|M1CAU1\|M1CAU1_SOLTU 26S protease regulatory subunit 6A homolog OS=Solanum tuberosum OX=4113 GN=102580368 PE=3 SV=1 | 15 | 56,3 | 47,482 | 323,31 | 5,83E+08 | 4,72E+08 | 8,10E-01 | DOWN | + | 1,40462 | 0,039 | 0,298195 | 2,45884 |
| M1D576 | 102587729 | tr\|M1D576\|M1D576_SOLTU formate--tetrahydrofolate ligase OS=Solanum tuberosum OX=4113 GN=102587729 PE=3 SV=1;tr\|M1D577\|M1D577_SOLTU formate--tetrahydrofolate ligase OS=Solanum tuberosum OX=4113 GN=102587729 PE=4 SV=1 | 15 | 39,3 | 67,299 | 124,08 | 2,58E+08 | 2,02E+08 | 7,83E-01 | DOWN | + | 1,90518 | 0,012 | 0,350266 | 3,20916 |
| M1CKC5 | 102605742 | tr\|M1CKC5\|M1CKC5_SOLTU Glyceraldehyde-3-phosphate dehydrogenase OS=Solanum tuberosum OX=4113 GN=102605742 PE=3 SV=1 | 16 | 56,1 | 36,643 | 323,31 | 2,28E+08 | 1,64E+08 | 7,19E-01 | DOWN | + | 2,1824 | 0,007 | 0,462886 | 3,64193 |
| M1BAA6 | 102580488 | tr\|M1BAA6\|M1BAA6_SOLTU Serine hydroxymethyltransferase OS=Solanum tuberosum OX=4113 GN=102580488 PE=3 SV=1 | 17 | 54,6 | 52,044 | 323,31 | 7,38E+08 | 6,09E+08 | 8,25E-01 | DOWN | + | 1,68502 | 0,021 | 0,267178 | 2,87553 |
| M0ZRG6 | 102577751 | tr\|M0ZRG6\|M0ZRG6_SOLTU Rubisco subunit binding-protein beta subunit, rubb OS=Solanum tuberosum OX=4113 GN=102577751 PE=3 SV=1 | 17 | 42,5 | 64,312 | 161,3 | 8,04E+07 | 5,72E+07 | 7,11E-01 | DOWN | + | 2,30864 | 0,005 | 0,484597 | 3,84486 |
| M0ZV40 | 102593980 | tr\|M0ZV40\|M0ZV40_SOLTU Vacuolar proton pump subunit B OS=Solanum tuberosum OX=4113 GN=102593980 PE=3 SV=1;tr\|M0ZV38\|M0ZV38_SOLTU Vacuolar proton pump subunit B OS=Solanum tuberosum OX=4113 GN=102593980 PE=3 SV=1 | 18 | 53,3 | 54,242 | 323,31 | 1,49E+09 | 1,19E+09 | 7,99E-01 | DOWN | + | 2,69221 | 0,002 | 0,322327 | 4,48902 |
| Q3YJS9 | pat3-k1 | sp\|Q3YJS9\|PT3K1_SOLTU Probable inactive patatin-3-Kuras 1 OS=Solanum tuberosum OX=4113 GN=pat3-k1 PE=1 SV=1 | 18 | 65 | 41,192 | 323,31 | 2,08E+10 | 1,29E+10 | 6,20E-01 | DOWN | + | 3,33063 | 0,000 | 0,682893 | 5,67616 |
| M0ZKX1 | 102594819 | tr\|M0ZKX1\|M0ZKX1_SOLTU RuBisCO large subunit-binding protein subunit alpha, chloroplastic OS=Solanum tuberosum OX=4113 GN=102594819 PE=3 SV=1 | 19 | 46,8 | 61,897 | 323,31 | 5,37E+08 | 4,55E+08 | 8,47E-01 | DOWN | + | 2,63572 | 0,002 | 0,237997 | 4,39129 |
| M1BJD2 | 102600114 | tr\|M1BJD2\|M1BJD2_SOLTU Cell division cycle protein 48 OS=Solanum tuberosum OX=4113 GN=102600114 PE=4 SV=1 | 19 | 34,7 | 90,054 | 81,386 | 3,78E+07 | 3,12E+07 | 8,25E-01 | DOWN | + | 2,25375 | 0,006 | 0,275466 | 3,75614 |
| M1C7V6 | 102577525 | tr\|M1C7V6\|M1C7V6_SOLTU Chaperonin-60 beta subunit OS=Solanum tuberosum OX=4113 GN=102577525 PE=3 SV=1;tr\|M1C7V8\|M1C7V8_SOLTU Chaperonin-60 beta subunit OS=Solanum tuberosum OX=4113 GN=102577525 PE=3 SV=1;tr\|M1C7V7\|M1C7V7_SOLTU Chaperonin-60 beta subunit OS | 20 | 46,7 | 61,549 | 323,31 | 8,88E+08 | 6,94E+08 | 7,82E-01 | DOWN | + | 2,45077 | 0,004 | 0,354554 | 4,07843 |
| M1B7H6 | 102582495 | tr\|M1B7H6\|M1B7H6_SOLTU Aspartic protease OS=Solanum tuberosum OX=4113 GN=102582495 PE=3 SV=1 | 21 | 48,6 | 55,163 | 323,31 | 3,12E+09 | 1,86E+09 | 5,96E-01 | DOWN | + | 1,93379 | 0,012 | 0,695074 | 3,2531 |
| M1D096 | 102599894 | tr\|M1D096\|M1D096_SOLTU ATP synthase subunit beta OS=Solanum tuberosum OX=4113 GN=102599894 PE=3 SV=1;tr\|M1D0F0\|M1D0F0_SOLTU ATP synthase subunit beta OS=Solanum tuberosum OX=4113 GN=102603549 PE=3 SV=1 | 22 | 60,8 | 59,568 | 323,31 | 5,17E+09 | 4,29E+09 | 8,30E-01 | DOWN | + | 2,00583 | 0,010 | 0,269978 | 3,36444 |
| M1AVI1 | 102589113 | tr\|M1AVI1\|M1AVI1_SOLTU Cell division control protein OS=Solanum tuberosum OX=4113 GN=102589113 PE=4 SV=1 | 23 | 39,5 | 89,488 | 323,31 | 5,09E+08 | 4,20E+08 | 8,25E-01 | DOWN | + | 1,87796 | 0,013 | 0,273655 | 3,16748 |
| M0ZSD6 | 102595943 | tr\|M0ZSD6\|M0ZSD6_SOLTU phosphopyruvate hydratase OS=Solanum tuberosum OX=4113 GN=102595943 PE=3 SV=1 | 24 | 76,4 | 47,845 | 323,31 | 5,39E+09 | 4,40E+09 | 8,16E-01 | DOWN | + | 2,46438 | 0,003 | 0,291632 | 4,10109 |
| M1B4C9 | 102588418 | tr\|M1B4C9\|M1B4C9_SOLTU Heat shock protein OS=Solanum tuberosum OX=4113 GN=102588418 PE=3 SV=1;tr\|M1B4C8\|M1B4C8_SOLTU Heat shock protein OS=Solanum tuberosum OX=4113 GN=102588418 PE=3 SV=1 | 26 | 56,6 | 71,888 | 323,31 | 3,74E+08 | 2,43E+08 | 6,50E-01 | DOWN | + | 4,5984 | 0,000 | 0,620155 | 8,62966 |
| M1BS78 | 102595780 | tr\|M1BS78\|M1BS78_SOLTU Wound-induced basic protein OS=Solanum tuberosum OX=4113 GN=102595780 PE=4 SV=1;tr\|M1ACX5\|M1ACX5_SOLTU Wound-induced basic protein OS=Solanum tuberosum OX=4113 PE=4 SV=1 | 1 | 23,4 | 5,4251 | 99,204 | 5,33E+07 | 1,00E+00 | 5,33E+07 | Only CTRL | + | 1,39744 | 0,040 | 15,7848 | 2,44824 |
| M1CVH2 | 102578172 | tr\|M1CVH2\|M1CVH2_SOLTU Localized to the inner membrane of the chloroplast OS=Solanum tuberosum OX=4113 GN=102578172 PE=4 SV=1;tr\|M1CVH3\|M1CVH3_SOLTU Localized to the inner membrane of the chloroplast OS=Solanum tuberosum OX=4113 GN=102578172 PE=4 SV=1 | 1 | 10,1 | 17,042 | 154,62 | 3,09E+07 | 1,00E+00 | 3,09E+07 | Only CTRL | + | 14,6238 | 0,000 | 24,8471 | 161,834 |
| M1D1J4 | 102578404 | tr\|M1D1J4\|M1D1J4_SOLTU Ubiquitin-fold modifier 1 OS=Solanum tuberosum OX=4113 GN=102578404 PE=3 SV=1;tr\|M1CUY6\|M1CUY6_SOLTU Ubiquitin-fold modifier 1 OS=Solanum tuberosum OX=4113 GN=102590609 PE=3 SV=1 | 2 | 43,9 | 10,229 | 26,763 | 2,99E+07 | 1,00E+00 | 2,99E+07 | Only CTRL | + | 2,40195 | 0,004 | 20,0767 | 3,99756 |
| M1CZL6 | 102605762 | tr\|M1CZL6\|M1CZL6_SOLTU Hsp20.1 protein OS=Solanum tuberosum OX=4113 GN=102605762 PE=3 SV=1 | 3 | 28,6 | 17,689 | 279,87 | 2,44E+08 | 1,00E+00 | 2,44E+08 | Only CTRL | + | 1,39768 | 0,040 | 17,115 | 2,44861 |
| M1CYG3 | 102593339 | tr\|M1CYG3\|M1CYG3_SOLTU Ectonucleotide pyrophosphatase/phosphodiesterase OS=Solanum tuberosum OX=4113 GN=102593339 PE=4 SV=1;tr\|M1CYG2\|M1CYG2_SOLTU Ectonucleotide pyrophosphatase/phosphodiesterase OS=Solanum tuberosum OX=4113 GN=102593339 PE=4 SV=1;tr\|M1CYG | 3 | 9,5 | 52,806 | 31,687 | 3,72E+07 | 1,00E+00 | 3,72E+07 | Only CTRL | + | 2,40295 | 0,004 | 20,3627 | 3,99921 |
| M1BP57 | 102579105 | tr\|M1BP57\|M1BP57_SOLTU ATP binding protein OS=Solanum tuberosum OX=4113 GN=102579105 PE=4 SV=1 | 3 | 15,1 | 31,878 | 30,85 | 3,26E+07 | 1,00E+00 | 3,26E+07 | Only CTRL | + | 1,39749 | 0,040 | 15,3679 | 2,44832 |
| M0ZT74 | 102601242 | tr\|M0ZT74\|M0ZT74_SOLTU Pyruvate dehydrogenase E1 component subunit alpha OS=Solanum tuberosum OX=4113 GN=102601242 PE=4 SV=1 | 3 | 13,2 | 48,776 | 32,073 | 1,06E+07 | 1,00E+00 | 1,06E+07 | Only CTRL | + | 2,40255 | 0,004 | 18,9031 | 3,99855 |
| M1C1E7 | 102602039 | tr\|M1C1E7\|M1C1E7_SOLTU Epoxide hydrolase OS=Solanum tuberosum OX=4113 GN=102602039 PE=4 SV=1 | 3 | 13,7 | 35,511 | 46,73 | 8,80E+06 | 1,00E+00 | 8,80E+06 | Only CTRL | + | 1,39721 | 0,040 | 14,2232 | 2,4479 |
| M1CVI5 | 102585121 | tr\|M1CVI5\|M1CVI5_SOLTU glutathione transferase OS=Solanum tuberosum OX=4113 GN=102585121 PE=3 SV=1 | 3 | 14,5 | 25,146 | 28,831 | 5,89E+06 | 1,00E+00 | 5,89E+06 | Only CTRL | + | 1,39731 | 0,040 | 13,8859 | 2,44806 |
| M1D7P9 | 102589664 | tr\|M1D7P9\|M1D7P9_SOLTU Adenylyl cyclase-associated protein OS=Solanum tuberosum OX=4113 GN=102589664 PE=3 SV=1 | 3 | 8,5 | 50,829 | 94,749 | 3,10E+06 | 1,00E+00 | 3,10E+06 | Only CTRL | + | 1,39688 | 0,040 | 13,3087 | 2,44741 |
| M0ZRU4 | 102599664 | tr\|M0ZRU4\|M0ZRU4_SOLTU IAA-amino acid hydrolase OS=Solanum tuberosum OX=4113 GN=102599664 PE=3 SV=1 | 3 | 12,1 | 48,927 | 37,48 | 2,63E+06 | 1,00E+00 | 2,63E+06 | Only CTRL | + | 1,39711 | 0,040 | 13,1828 | 2,44777 |
| M1BTZ0 | 102590612 | tr\|M1BTZ0\|M1BTZ0_SOLTU Nuclear RNA binding protein OS=Solanum tuberosum OX=4113 GN=102590612 PE=4 SV=1 | 4 | 16,1 | 39,176 | 27,8 | 1,66E+08 | 1,00E+00 | 1,66E+08 | Only CTRL | + | 1,3976 | 0,040 | 16,7754 | 2,44848 |
| M1AYY7 | 102582419 | tr\|M1AYY7\|M1AYY7_SOLTU Aminotransferase OS=Solanum tuberosum OX=4113 GN=102582419 PE=3 SV=1 | 4 | 9,1 | 51,666 | 31,672 | 2,38E+07 | 1,00E+00 | 2,38E+07 | Only CTRL | + | 1,39753 | 0,040 | 15,1011 | 2,44838 |
| M1D3H2 | 102597571 | tr\|M1D3H2\|M1D3H2_SOLTU Zinc finger protein OS=Solanum tuberosum OX=4113 GN=102597571 PE=3 SV=1 | 4 | 16,9 | 40,588 | 24,973 | 1,28E+07 | 1,00E+00 | 1,28E+07 | Only CTRL | + | 1,39745 | 0,040 | 14,5597 | 2,44826 |
| M1C8Y1 | 102587438 | tr\|M1C8Y1\|M1C8Y1_SOLTU Proteasome inhibitor OS=Solanum tuberosum OX=4113 GN=102587438 PE=3 SV=1;tr\|M1C8Y0\|M1C8Y0_SOLTU Proteasome inhibitor OS=Solanum tuberosum OX=4113 GN=102587438 PE=3 SV=1;tr\|M1C8X9\|M1C8X9_SOLTU Proteasome inhibitor OS=Solanum tuberosum | 5 | 18,8 | 32,302 | 59,107 | 2,37E+07 | 1,00E+00 | 2,37E+07 | Only CTRL | + | 1,39747 | 0,040 | 15,0907 | 2,44829 |
| M1D6F8 | 102584305 | tr\|M1D6F8\|M1D6F8_SOLTU EMB514 OS=Solanum tuberosum OX=4113 GN=102584305 PE=4 SV=1 | 5 | 21,7 | 25,559 | 29,839 | 9,10E+06 | 1,00E+00 | 9,10E+06 | Only CTRL | + | 1,3968 | 0,040 | 14,2242 | 2,4473 |
| M1CPV6 | 102585419 | tr\|M1CPV6\|M1CPV6_SOLTU methenyltetrahydrofolate cyclohydrolase OS=Solanum tuberosum OX=4113 GN=102585419 PE=3 SV=1;tr\|M1CPV5\|M1CPV5_SOLTU methenyltetrahydrofolate cyclohydrolase OS=Solanum tuberosum OX=4113 GN=102585419 PE=3 SV=1 | 6 | 22,7 | 32,162 | 35,272 | 1,37E+07 | 1,00E+00 | 1,37E+07 | Only CTRL | + | 1,39725 | 0,040 | 14,6042 | 2,44797 |
| M0ZVB0 | 102589018 | tr\|M0ZVB0\|M0ZVB0_SOLTU Oxidoreductase, 2OG-Fe(II) oxygenase family protein OS=Solanum tuberosum OX=4113 GN=102589018 PE=3 SV=1;tr\|M0ZVA9\|M0ZVA9_SOLTU Oxidoreductase, 2OG-Fe(II) oxygenase family protein OS=Solanum tuberosum OX=4113 GN=102589018 PE=4 SV=1 | 6 | 28,4 | 31,305 | 67,149 | 4,49E+06 | 1,00E+00 | 4,49E+06 | Only CTRL | + | 1,39738 | 0,040 | 13,6573 | 2,44816 |
| M0ZXA4 | 102598129 | tr\|M0ZXA4\|M0ZXA4_SOLTU Superoxide dismutase copper chaperone OS=Solanum tuberosum OX=4113 GN=102598129 PE=3 SV=1;tr\|M0ZXA2\|M0ZXA2_SOLTU Superoxide dismutase copper chaperone OS=Solanum tuberosum OX=4113 GN=102598129 PE=3 SV=1 | 7 | 38,9 | 22,46 | 49,061 | 6,63E+06 | 1,00E+00 | 6,63E+06 | Only CTRL | + | 2,40208 | 0,004 | 18,3526 | 3,99777 |
| M1CAS5 | 102604599 | tr\|M1CAS5\|M1CAS5_SOLTU Luminal binding protein OS=Solanum tuberosum OX=4113 GN=102604599 PE=3 SV=1 | 8 | 13,6 | 74,678 | 30,724 | 5,53E+06 | 1,00E+00 | 5,53E+06 | Only CTRL | + | 1,39728 | 0,040 | 13,8304 | 2,44801 |
| M1CLW5 | 102582462 | tr\|M1CLW5\|M1CLW5_SOLTU Pyruvate kinase OS=Solanum tuberosum OX=4113 GN=102582462 PE=3 SV=1 | 9 | 22,4 | 57,545 | 31,621 | 9,14E+06 | 1,00E+00 | 9,14E+06 | Only CTRL | + | 1,39679 | 0,040 | 14,2273 | 2,44729 |
| M1C6I0 | 102603407 | tr\|M1C6I0\|M1C6I0_SOLTU asparagine--tRNA ligase OS=Solanum tuberosum OX=4113 GN=102603407 PE=3 SV=1 | 5 | 10 | 64,683 | 30,313 | 1,00E+00 | 3,68E+06 | 3,68E+06 | ONLY LED | + | 1,39737 | 0,040 | -13,4863 | -2,44815 |
| M1BJW9 | 102601645 | tr\|M1BJW9\|M1BJW9_SOLTU glutamate--tRNA ligase OS=Solanum tuberosum OX=4113 GN=102601645 PE=3 SV=1 | 5 | 7,3 | 84,199 | 43,513 | 1,00E+00 | 2,51E+06 | 2,51E+06 | ONLY LED | + | 1,39731 | 0,040 | -13,1536 | -2,44806 |
| M0ZSJ3 | 102580709 | tr\|M0ZSJ3\|M0ZSJ3_SOLTU Nucleolar protein 56 OS=Solanum tuberosum OX=4113 GN=102580709 PE=3 SV=1;tr\|M0ZSJ4\|M0ZSJ4_SOLTU Nucleolar protein 56 OS=Solanum tuberosum OX=4113 GN=102580709 PE=3 SV=1;tr\|M0ZSJ5\|M0ZSJ5_SOLTU Nucleolar protein 56 OS=Solanum tuberosum | 10 | 37,7 | 43,564 | 76,021 | 1,00E+00 | 1,23E+08 | 1,23E+08 | ONLY LED | + | 1,39766 | 0,040 | -16,5234 | -2,44858 |
| M1CZH5 | 102589455 | tr\|M1CZH5\|M1CZH5_SOLTU NADH dehydrogenase OS=Solanum tuberosum OX=4113 GN=102589455 PE=3 SV=1 | 3 | 37,8 | 11,098 | 30,792 | 9,13E+06 | 2,54E+07 | 2,78E+00 | UP | + | 1,44816 | 0,036 | -14,9325 | -2,52318 |
| M0ZHU5 | 102596553 | tr\|M0ZHU5\|M0ZHU5_SOLTU Osmotin OS=Solanum tuberosum OX=4113 GN=102596553 PE=4 SV=1 | 3 | 12,7 | 27,176 | 20,391 | 3,72E+07 | 6,67E+07 | 1,79E+00 | UP | + | 1,66421 | 0,022 | -0,873792 | -2,84434 |
| M1BC63 | 102577562 | tr\|M1BC63\|M1BC63_SOLTU Acyl-[acyl-carrier-protein] desaturase OS=Solanum tuberosum OX=4113 GN=102577562 PE=3 SV=1;sp\|P46253\|STAD_SOLTU Stearoyl-[acyl-carrier-protein] 9-desaturase, chloroplastic OS=Solanum tuberosum OX=4113 PE=2 SV=1;tr\|M0ZKA3\|M0ZKA3_SOLTU | 6 | 18,1 | 44,636 | 111,5 | 5,02E+06 | 2,16E+07 | 4,30E+00 | UP | + | 1,52535 | 0,030 | -15,0509 | -2,63748 |
| M1D4F7 | 102586126 | tr\|M1D4F7\|M1D4F7_SOLTU Phospholipase A1 OS=Solanum tuberosum OX=4113 GN=102586126 PE=3 SV=1 | 6 | 21,6 | 44,994 | 144,27 | 7,13E+07 | 1,29E+08 | 1,81E+00 | UP | + | 2,00415 | 0,010 | -0,928988 | -3,36183 |
| M1AFU6 | 102585477 | tr\|M1AFU6\|M1AFU6_SOLTU Polygalacturonase inhibiting protein OS=Solanum tuberosum OX=4113 GN=102585477 PE=3 SV=1 | 7 | 22,5 | 37,913 | 49,922 | 3,75E+07 | 4,59E+07 | 1,22E+00 | UP | + | 1,72264 | 0,019 | -0,294735 | -2,93203 |
| P93787 | 35G | tr\|P93787\|P93787_SOLTU 14-3-3 protein OS=Solanum tuberosum OX=4113 GN=35G PE=2 SV=2 | 8 | 39,8 | 28,201 | 75,327 | 1,82E+07 | 2,71E+07 | 1,49E+00 | UP | + | 1,3334 | 0,046 | -0,600142 | -2,35375 |
| M1BQT9 | 102591897 | tr\|M1BQT9\|M1BQT9_SOLTU Protease C56 OS=Solanum tuberosum OX=4113 GN=102591897 PE=3 SV=1;tr\|M1BQU0\|M1BQU0_SOLTU Protease C56 OS=Solanum tuberosum OX=4113 GN=102591897 PE=3 SV=1 | 10 | 41,5 | 41,865 | 168,27 | 1,14E+08 | 1,44E+08 | 1,26E+00 | UP | + | 1,46679 | 0,034 | -0,336002 | -2,55073 |
| M1D0T5 | 102579479 | tr\|M1D0T5\|M1D0T5_SOLTU H(+)-exporting diphosphatase OS=Solanum tuberosum OX=4113 GN=102579479 PE=3 SV=1;tr\|M1B4C0\|M1B4C0_SOLTU H(+)-exporting diphosphatase OS=Solanum tuberosum OX=4113 GN=102583477 PE=3 SV=1;tr\|M1ADJ8\|M1ADJ8_SOLTU H(+)-exporting diphosphat | 12 | 21,4 | 80,236 | 244,07 | 1,90E+08 | 4,31E+08 | 2,27E+00 | UP | + | 1,85966 | 0,014 | -1,19991 | -3,13955 |
| M1B8A0 | 102588806 | tr\|M1B8A0\|M1B8A0_SOLTU Protein tyrosine phosphatase OS=Solanum tuberosum OX=4113 GN=102588806 PE=4 SV=1 | 12 | 48,1 | 41,564 | 160,52 | 2,50E+08 | 3,14E+08 | 1,26E+00 | UP | + | 1,77777 | 0,017 | -0,337883 | -3,01519 |
| M1CZK7 | 102601164 | tr\|M1CZK7\|M1CZK7_SOLTU Sorting and assembly machinery (Sam50) protein OS=Solanum tuberosum OX=4113 GN=102601164 PE=4 SV=1;tr\|M1CZK6\|M1CZK6_SOLTU Sorting and assembly machinery (Sam50) protein OS=Solanum tuberosum OX=4113 GN=102601164 PE=4 SV=1 | 12 | 20,6 | 88,755 | 123,71 | 1,30E+07 | 1,60E+07 | 1,23E+00 | UP | + | 1,58605 | 0,026 | -0,294019 | -2,72768 |
| M1AMY7 | 102594586 | tr\|M1AMY7\|M1AMY7_SOLTU Cysteine protease inhibitor 1 OS=Solanum tuberosum OX=4113 GN=102595571 PE=3 SV=1;tr\|M1AMY4\|M1AMY4_SOLTU Cysteine protease inhibitor 1 OS=Solanum tuberosum OX=4113 GN=102594586 PE=3 SV=1;tr\|M1AMY9\|M1AMY9_SOLTU Cysteine protease inhib | 13 | 42,8 | 24,774 | 323,31 | 5,54E+09 | 9,34E+09 | 1,69E+00 | UP | + | 2,36441 | 0,004 | -0,745529 | -3,93584 |
| M1D357 | 102594404 | tr\|M1D357\|M1D357_SOLTU Vacuolar sorting receptor protein PV72 OS=Solanum tuberosum OX=4113 GN=102594404 PE=3 SV=1 | 14 | 29 | 69,188 | 107,44 | 6,35E+07 | 1,05E+08 | 1,65E+00 | UP | + | 1,97015 | 0,011 | -0,743971 | -3,30916 |
| M1CV13 | PFP-ALPHA | tr\|M1CV13\|M1CV13_SOLTU Pyrophosphate--fructose 6-phosphate 1-phosphotransferase subunit alpha OS=Solanum tuberosum OX=4113 GN=PFP-ALPHA PE=3 SV=1 | 15 | 34,7 | 67,351 | 156,25 | 6,82E+07 | 1,04E+08 | 1,52E+00 | UP | + | 1,40698 | 0,039 | -0,637962 | -2,46233 |
| M1B4H0 | 102599872 | tr\|M1B4H0\|M1B4H0_SOLTU Ribonuclease OS=Solanum tuberosum OX=4113 GN=102599872 PE=4 SV=1 | 16 | 22,3 | 107,64 | 193,67 | 5,92E+07 | 9,35E+07 | 1,58E+00 | UP | + | 2,18821 | 0,006 | -0,673056 | -3,65119 |
| M0ZT40 | SS16 | tr\|M0ZT40\|M0ZT40_SOLTU Sucrose synthase OS=Solanum tuberosum OX=4113 GN=102577594 PE=3 SV=1;sp\|P10691\|SUS1_SOLTU Sucrose synthase OS=Solanum tuberosum OX=4113 PE=2 SV=1 | 31 | 52 | 92,47 | 323,31 | 5,98E+08 | 2,22E+09 | 3,71E+00 | UP | + | 3,72009 | 0,000 | -1,89481 | -6,48798 |
| Q43190 | LOX1.4 | sp\|Q43190\|LOX14_SOLTU Probable linoleate 9S-lipoxygenase 4 OS=Solanum tuberosum OX=4113 GN=LOX1.4 PE=2 SV=1 | 46 | 71,9 | 96,968 | 323,31 | 7,92E+08 | 1,02E+09 | 1,29E+00 | UP | + | 1,36593 | 0,043 | -0,391349 | -2,40175 |
| O22508 | LOX1.8 | sp\|O22508\|LOX18_SOLTU Probable linoleate 9S-lipoxygenase 8 OS=Solanum tuberosum OX=4113 GN=LOX1.8 PE=2 SV=1 | 51 | 72,9 | 97,019 | 323,31 | 9,93E+09 | 1,45E+10 | 1,46E+00 | UP | + | 1,6004 | 0,025 | -0,573457 | -2,74904 |

**Table S7.** Annotated and identified metabolites differentially represented in potato tubers under LED supplementation compared with sunlight, including molecular formula, m/z values, and statistical parameters across ionization modes.

| **Compound classes** | **Name** | **Formula** | **Calc. MW** | **[M-H]-** | **[M+H]+** | **RT [min]** | **Mass Error (ppm)** | **Log2 Fold Change: (LED) / (CTRL)** | **P-value: (LED) / (CTRL)** | **Source** | **Over-represented in LED or Control samples** |
| --- | --- | --- | --- | --- | --- | --- | --- | --- | --- | --- | --- |
| **Polyphenols** |  |  |  |  |  |  |  |  |  |  |  |
| Hydroxycinnamic acid | caffeoyl hexose | C15 H18 O9 | 342.0949 | 341.0877 |  | 2.84 | -0.43 | 0.3 | 0.691154739 | potato tuber |  |
| Hydroxycinnamic acid | caffeoyl hexose | C15 H18 O9 | 342.0952 | 341.0879 |  | 2.75 | 1.83 | -0.36 | 0.014754855 | potato tuber | x |
| Hydroxycinnamic acid | chlorogenic acid-cryptochlorogenic acid | C16 H18 O9 | 354.0947 | 353.0875 |  | 3.17 | -0.98 | -0.22 | 0.017998948 | potato tuber | x |
| Hydroxycinnamic acid | neochlorogenic acid | C16 H18 O9 | 354.0951 | 353.0878 |  | 2.47 | -0.04 | -0.74 | 2.04422E-05 | potato tuber | x |
| Hydroxycinnamic acid | *cis* chlorogenic acid | C16 H18 O9 | 354.0951 | 353.0879 |  | 3.56 | 0.11 | -0.26 | 0.008353684 | potato tuber | x |
| Hydroxycinnamic acid | feruloyl hexose | C16 H20 O9 | 356.1108 | 355.1035 |  | 3.66 | 0.18 | -0.56 | 0.000339968 | potato tuber | x |
| Hydroxycinnamic acid | 3-O-feruloyl-D-quinic acid | C17 H20 O9 | 368.1106 | 367.1034 |  | 4.16 | -0.24 | -0.16 | 0.077767495 | potato tuber |  |
| Hydroxycinnamic acid | 4-O-feruloyl-D-quinic acid | C17 H20 O9 | 368.1107 | 367.1034 |  | 4.06 | -0.05 | -0.76 | 0.00052903 | potato tuber |  |
| Hydroxycinnamic acid | 5-O-feruloyl-D-quinic acid | C17 H20 O9 | 368.1107 | 367.1035 |  | 4.43 | 0.02 | -0.15 | 0.215052579 | potato tuber | x |
| Hydroxycinnamic acid | 4, 5 dicaffeoyl quinic acid | C25 H24 O12 | 516.1270 | 515.1197 |  | 6.02 | 0.44 | -0.09 | 0.185360803 | potato tuber |  |
| Hydroxycinnamic acid | 3,5 dicaffeoyl quinic acid | C25 H24 O12 | 516.1270 | 515.1198 |  | 5.81 | 0.48 | -0.33 | 0.005405256 | potato tuber | x |
| Hydroxybenzoic acid | vanillic acid hexoside | C13 H16 O9 | 316.0798 | 315.0725 |  | 1.56 | 1.02 | -0.39 | 0.963792975 |  |  |
| Hydroxybenzoic acid | 1-O-vanilloyl-beta-D-glucose | C14 H18 O9 | 330.0954 | 329.0881 |  | 1.66 | 0.86 | -0.34 | 0.00127419 |  |  |
| **Polyamine Derivatives** |  |  |  |  |  |  |  |  |  |  |  |
| phenolic amine | N-Caffeoylputrescine | C13 H18 N2 O3 | 250.1319 |  | 251.1390 | 1.84 | -0.04 | 1.19 | 0.033980641 | potato tuber | x |
| phenolic amine | Feruloyl putrescine | C14 H20 N2 O3 | 264.1473 |  | 265.1546 | 3.09 | -0.30 | 0.56 | 0.015867183 | potato tuber | x |
| phenolic amine | Di(dihydrocaffeoyl) spermidine | C25 H35 N3 O6 | 473.2526 | 472.2453 |  | 3.51 | 0.02 | 0.17 | 0.423534872 | potato tuber |  |
| phenolic amine | Tris(dihydrocaffeoyl) spermidine | C34 H43 N3 O9 | 637.3002 | 636.2930 |  | 6.07 | 0.49 | 0.28 | 0.446884904 | potato tuber |  |
| phenolic amine | Tris(dihydrocaffeoyl) spermine | C37H50N4O9 | 694.3581 | 693.3508 |  | 4.76 | 1.96 | 0.21 | 0.440687438 | potato tuber |  |
| phenolic amine | N1,N14-di(dihydrocaffeoyl) spermine (Kukoamine B) | C28 H42 N4 O6 | 530.3104 | 529.3031 |  | 2.84 | -0.12 | 0.21 | 0.474425641 | potato tuber |  |
| **Free Polyamine** | Spermidine | C7 H19 N3 | 145.15778 |  | 146.1651 | 0.60 | -0.83 | 0 | 0.605034535 | potato |  |
|  | Spermine | C10 H26 N4 | 202.21559 |  | 203.2229 | 0.59 | -0.77 | -0.38 | 0.239278717 | potato |  |
| **Amino acids and derivatives** | Isoleucine | C6 H13 N O2 | 131.0945 |  | 132.1018 | 0.91 | -0.89 | -0.37 | 0.115544871 | potato tuber |  |
|  | Methionine | C5 H11 N O2 S | 149.0510 |  | 150.0582 | 0.86 | -0.61 | -0.05 | 0.539769416 | potato tuber |  |
|  | Lysine | C6 H14 N2 O2 | 146.1054 |  | 147.1128 | 0.64 | -0.83 | 0 | 0.180862829 | potato tuber |  |
|  | Phenylalanine | C9 H11 N O2 | 165.0788 |  | 166.0861 | 1.43 | -0.85 | -0.05 | 0.623681728 | potato tuber |  |
|  | Tryptophan | C11 H12 N2 O2 | 204.0897 |  | 205.0972 | 2.61 | -0.70 | -0.32 | 0.02142086 | potato tuber | x |
|  | Asparagine | C4 H8 N2 O3 | 132.0534 |  | 133.0606 | 0.70 | -1.11 | 0.38 | 0.19227747 | potato tuber |  |
|  | Aspartic acid | C4 H7 N O4 | 133.0374 |  | 134.0447 | 0.70 | -1.02 | -0.16 | 0.13941816 | potato tuber |  |
|  | Tyrosine | C9 H11 N O3 | 181.0737 |  | 182.0810 | 0.90 | -0.86 | -0.12 | 0.276510593 | potato tuber |  |
|  | L-Glutamic acid | C5 H9 N O4 | 147.0530 |  | 148.0603 | 0.70 | -0.77 | -0.65 | 0.34367897 | potato tuber |  |
|  | Arginine | C6 H14 N4 O2 | 174.1115 |  | 175.1188 | 0.65 | -1.20 | 0.36 | 0.056171293 |  |  |
|  | Glutamine | C5 H10 N2 O3 | 146.0690 |  | 147.0764 | 0.70 | -0.85 | 0.43 | 0.000688759 |  | x |
|  | N-Acetyl-L-leucine | C8 H15 N O3 | 173.1050 |  | 174.1123 | 0.78 | -0.92 | 0.27 | 0.031930181 |  | x |
|  | N-Acetylornithine | C7 H14 N2 O3 | 174.1002 |  | 175.1075 | 0.73 | -1.17 | -0.03 | 0.42786167 | potato leaves |  |
|  | N6-Acetyl-L-lysine | C8 H16 N2 O3 | 188.1159 |  | 189.1232 | 0.76 | -1.02 | -0.16 | 0.05199121 | potato leaves |  |
|  | Ornithine | C5 H12 N2 O2 | 132.0898 |  | 131.0825 | 0.654 | -0.54 | 0.65 | 0.000352848 | potato tuber |  |
|  | L-Histidine | C6 H9 N3 O2 | 155.06938 | 154.06211 |  | 0.659 | -0.59 |  |  | potato leaves |  |
|  | N6,N6,N6-Trimethyl-L-lysine | C9 H20 N2 O2 | 188.1523 |  | 189.1595 | 0.67 | -1.16 | -0.13 | 0.23973189 |  |  |
|  | N,N-dimethylarginine | C8 H18 N4 O2 | 202.1430 |  | 203.1500 | 0.70 | 0.28 | 0.59 | 7.93616E-06 | potato leaves | x |
| dipeptide | Val-Glu | C10 H18 N2 O5 | 246.1216 |  | 247.1289 | 1.15 | 0.21 | 0.42 | 0.016115304 |  |  |
| tripeptide | L-Glutathione (reduced) | C10 H17 N3 O6 S | 307.08362 | 306.07635 |  | 0.87 | -0.6 | -0.75 | 0.000925063 | potato tuber | x |
| L-alpha amino acid | L-Pyroglutamic acid | C5 H7 N O3 | 129.0426 |  | 130.0498 | 0.93 | -0.35 | 0.73 | 0.00101332 | potato tuber | x |
| **Glycoalkaloids** | dehydrochaconine | C45 H71 N O14 | 849.4874 |  | 850.4946 | 8.24 | -0.13 | -0.51 | 0.002920258 | potato tuber | x |
|  | dehydrochaconine | C45 H71 N O14 | 849.4875 |  | 850.4948 | 7.28 | 0.06 | -0.52 | 0.000720985 | potato tuber | x |
|  | alpha chaconine | C45 H73 N O14 | 851.5017 |  | 852.5090 | 8.49 | -1.64 | -0.39 | 0.003925024 | potato tuber | x |
|  | chaconine isomer | C45 H73 N O14 | 851.5023 |  | 852.5095 | 8.39 | -1.00 | -0.54 | 0.002352027 | potato tuber | x |
|  | solanidenone chacotriose | C45 H71 N O15 | 865.4813 |  | 866.4886 | 8.38 | -1.27 | -1.12 | 1.40807E-06 | potato tuber |  |
|  | solanidadienol chacotriose | C45 H71 N O15 | 865.4823 |  | 866.4896 | 6.48 | -0.08 | -1.19 | 5.18733E-05 | potato tuber | x |
|  | solanidadiene solatriose | C45 H71 N O15 | 865.4824 |  | 866.4896 | 7.13 | 0.00 | -0.47 | 0.013640465 | potato tuber | x |
|  | solanidadienol chacotriose isomer | C45 H71 N O15 | 865.4825 |  | 866.4898 | 6.27 | 0.14 | 0.33 | 0.664690546 | potato tuber |  |
|  | alpha solanine | C45 H73 N O15 | 867.4966 |  | 868.5039 | 8.41 | -1.59 | -0.36 | 0.008344726 | potato tuber | x |
|  | solanine isomer | C45 H73 N O15 | 867.4974 |  | 868.5047 | 8.29 | -0.67 | -0.63 | 0.004990041 | potato tuber | x |
|  | solanidadiene solatriose | C45 H73 N O15 | 867.4978 |  | 868.5051 | 8.72 | -0.23 | -0.26 | 0.027046116 | potato tuber | x |
|  | solanidenol chacotriose | C45 H73 N O15 | 867.4979 |  | 868.5052 | 7.10 | -0.16 | 0.68 | 0.2418857 | potato tuber |  |
|  | solanidenol chacotriose | C45 H73 N O15 | 867.4981 |  | 868.5053 | 5.30 | 0.04 | 0.06 | 0.606260319 | potato tuber |  |
|  | solanidadienol solatriose | C45 H71 N O16 | 881.4772 |  | 882.4845 | 6.36 | -0.06 | -1.17 | 5.59523E-05 | potato tuber | x |
| **Organic acids** |  |  |  |  |  |  |  |  |  |  |  |
| dicarboxylic acid | α-Ketoglutaric acid | C5 H6 O5 | 146.0214 |  | 147.0287 | 0.82 | -0.74 | -0.07 | 0.32462798 | potato tuber | x |
| piperidinemonocarboxylic acid | Pipecolic acid | C6 H11 N O2 | 129.0789 |  | 130.0861 | 0.80 | -0.94 | 0.42 | 0.016115304 | potato leaves | x |
|  | Citric acid | C6 H8 O7 | 192.0268 | 191.0195 |  | 0.82 | -1.01 | 0.02 | 0.755466389 | potato tuber |  |
|  | Quinic acid | C7 H12 O6 | 192.0633 | 191.0560 |  | 0.73 | -0.73 | 0.01 | 0.855304616 | potato tuber |  |
|  | Malic acid | C4 H6 O5 | 134.0215 | 133.0142 |  | 0.91 | -0.25 | 0.19 | 0.001177015 |  |  |
|  |  |  |  |  |  |  |  |  |  |  |  |
|  |  |  |  |  |  |  |  |  |  |  |  |
| **Sugars** | beta-Maltose | C12 H22 O11 | 342.1158 |  | 343.1235 | 0.69 | -0.06 | -0.19 | 0.042315807 |  | x |
|  | D-(+)-Xylose | C5 H10 O5 | 150.0527 | 149.0454 |  | 0.73 | -0.69 | 0.24 | 0.036645961 |  | x |
|  | D-(+)-Glucose | C6 H12 O6 | 180.0633 | 179.0560 |  | 0.7 | -0.79 | -0.09 | 0.645016861 |  |  |
| **Other** |  |  |  |  |  |  |  |  |  |  |  |
| Quaternary ammonium compound | Acetylcholine | C7 H15 N O2 | 145.1102 |  | 146.1175 | 0.86 | -0.60 | 0.51 | 0.007902228 |  | x |
| Vitamin | 5'-O-beta-D-Glucosylpyridoxine | C14 H21 N O8 | 331.1265 |  | 332.1337 | 0.89 | -0.76 | -0.39 | 0.016329517 |  | x |
| Vitamin | (+)-Riboflavin | C17 H20 N4 O6 | 376.1382 |  | 377.1455 | 4.06 | -0.28 | -0.39 | 0.016329517 |  | x |
| Vitamin | Ascorbic acid | C6 H8 O6 | 176.0320 | 175.0247 |  | 0.77 | -0.75 | -0.23 | 0.000896198 | potato tuber | x |
| alkaloids and derivatives | Trigonelline | C7 H7 N O2 | 137.0475 |  | 138.0548 | 0.75 | -1.07 | 0.45 | 3.73428E-06 |  | x |
| lipid | (10E,15Z)-9,12,13-Trihydroxy-10,15-octadecadienoic acid | C18 H32 O5 | 328.23 | 327.22 |  | 8.951 | 0.62 | 0.21 | 0.123510366 | potato tuber |  |

# 3 Supplemental methods

## 3.1 RNA extraction, library preparation and sequencing

Total RNA was extracted from six biological samples of Solanum tuberosum tubers, consisting of three replicates for the control condition and three for the LED condition. RNA extraction was performed using the Spectrum™ Plant Total RNA Kit (Sigma-Aldrich), following the manufacturer’s instructions. RNA quantity and purity were assessed using a NanoDrop 2000 spectrophotometer (Thermo Fisher Scientific, Waltham, MA, USA). RNA integrity was confirmed by agarose gel electrophoresis. Only RNA samples that passed quality control were used for library preparation. Libraries were prepared from high-quality RNA and sequenced on an Illumina NovaSeq 6000 platform, generating paired-end reads of 150 base pairs (2 × 150 bp).

## 3.2 RNA-seq data preprocessing, mapping, and differential expression analysis

The raw sequencing reads were processed with **fastp (Chen et al., 2018)** to trim adapter sequences and remove low-quality bases. This step yielded a total of 222.8 million clean reads across six samples, with an average of 37 million reads per sample. High-quality reads were mapped to the Solanum tuberosum reference genome (Stuberosum 686 v6.1) using **STAR (Dobin et al., 2013)**, achieving mapping rates between 86.36% and 88.19%. Strand specificity was confirmed as reverse-stranded using **RSeQC (Wang et al., 2012)**. Gene expression was quantified with **featureCounts (Liao et al., 2014)**. Genes with low expression levels were filtered using **HTSFilter (Rau et al., 2013)**, with an optimal threshold of s = 13.43. This retained 22,485 genes out of 32,917 detected (68.3%). Functional annotation of differentially expressed genes (DEGs) was inferred based on reciprocal best BLASTP hits (RBH) between *Solanum tuberosum* (DM v6.1) and *Arabidopsis thaliana* (TAIR10) which allowed us to identify homology for some genes. BLAST was performed using NCBI BLAST+ with an E-value cutoff of 1e‑5, retaining only high-confidence 1:1 orthologs. Gene Ontology (GO) and Kyoto Encyclopedia of Genes and Genomes (KEGG) pathway enrichment analyses were performed using the clusterProfiler R package (Yu et al., 2012), with a significant cutoff of p.adjust < 0.05. Only GO terms and KEGG pathways with at least three differentially expressed genes were considered. Differential gene expression between the LED and control conditions was assessed using the edgeR package (Robinson et al., 2010), applying a false discovery rate (FDR) cutoff of 0.05.

## 3.3 Proteomic analysis

### 3.3.1 Protein Extraction

Proteins were extracted from lyophilized tubers using Li et al. (2015) procedure, with modifications. Four independent biological replicates were analyzed in comparison for each light treatment. One gram of powder tissue was mixed with 0.01 g polyvinylpyrrolidone (Sigma-Aldrich) and 10 ml extraction buffer (0.7 M sucrose, 0.1 M KCl, 0.5 M Tris–HCl, 50 mM EDTA, 40 mM DTT, pH 8.5, supplemented with a protease inhibitor mix for plant tissues (Sigma-Aldrich). The mixture was homogenized (Ultra-Turrax, IKA, Werke GmbH Germany) with 5 cycles (20,000 rpm) of 10 sec, and 20-sec pauses on ice. Afterward, an equal volume of saturated phenol in 0.5M Tris-HCl, pH 8.1 (Sigma-Aldrich) was added, and the solution was incubated on a shaker for 20 min, at 4°C. The samples were centrifuged at 10,000 g for 10 min, at 4°C. The phenolic phase was recovered carefully to avoid contact with the interphase and poured into a new tube. This phenol phase was then back-extracted with 3 mL of resuspension buffer. All samples were shaken for 3 min and then vortexed. The phenolic phase was further recovered by centrifugation at 10000 g for 10 min, at 4°C. Proteins were precipitated overnight at −20 °C with six vol of ice-cold 0.1 M methanolic ammonium acetate, washed twice with ice-cold methanol and once with cold acetone containing 20 mM DTT, then air-dried. Protein pellets were dissolved in 1 mL of 8 M urea, 50 mM triethylammonium bicarbonate (TEAB), pH 8.5, vortexed, and incubated overnight at 30 °C under gentle shaking. Samples were then centrifuged at 12 000 × *g* for 5 min at 4 °C), and the supernatant was collected.

To reduce disulfide bonds, 100 µL of 100 mM DTT was added to each supernatant, followed by incubation at 60 ◦C for 1 h. Subsequently, 300 µL of 120 mM IAM was added, and the mixture was kept at room temperature in the dark for 1 h. Proteins were precipitated by adding five volumes of acetone and incubating overnight at −20 °C. After centrifugation at 12 000 × *g*, for 5 min, at 4 °C, the supernatant was discarded, and the protein pellet was dried briefly. Protein concentration was determined using the Bio-Rad Protein Assay (Bio-Rad Hercules, CA, USA), following the manufacturer’s instructions.

For each sample, 100 μg of protein extract was digested with 2 µL trypsin (1 mg/mL) at 37 ◦C overnight. The digestion was halted by placing the samples on ice for 10 min. The resulting peptide solution was dried, reconstituted in 50 µL of 50% v/v acetonitrile (ACN) containing 0.1% v/v trifluoroacetic acid (TFA), and desalted with ZipTip™ C18 tips (Millipore).

### 3.3.2 Protein identification and relative quantitation

Label-free proteomic analysis was performed, in three technical replicates, on the selected samples. Peptide mixtures were analyzed by means of a nanoUltraHighPressure liquid chromatography (nUHPLC)-electrospray (ESI)-Q-Orbitrap-MS/MS platform consisting of a Vanquish-Neo nano-chromatographer (Thermo Fisher Scientific) linked to an Exploris 480 mass spectrometer (Thermo Fisher Scientific) through an easy-spray ion source. Peptides were loaded on an EASY-Spray C18 column (250 mm × 75 μm ID, 2 μm particles, 100 Å pore size) (Thermo Fisher Scientific), and eluted with a gradient of solvent B (19.92/80/0.08 *v/v/v* water/acetonitrile/formic acid) in solvent A (99.9/0.1 *v/v* water/formic acid), at a flow rate of 250 nL/min. The gradient of solvent B started at 6%, increased to 31% over 90 min, increased to 50% over 5 min, increased to 95% over 5 min, remained at 95% for 8 min, and finally returned to 6% for equilibrating step. The mass spectrometer operated in data-dependent acquisition mode collecting MS spectra of positive ions within a scan *m/z* range of 375-1200, using a nominal resolution of 120,000 full width at half maximum (FWHM), an automatic gain control (AGC) target of 3 x 106 ions, a maximum injection time (IT) of 50 ms, and a dynamic exclusion value of 45 s, which was followed by MS/MS scans of the 20 most abundant ions. MS/MS spectra were acquired using a normalized collision energy (HCD) of 30%, a resolution of 15,000 FWHM, an AGC target of 2 x 105 ions, and a maximum IT of 100 ms.

### 3.3.3 Bioinformatic analysis of proteomic data

Proteomic data were processed using MaxQuant software (version v2.7.5.0) with the Andromeda search engine, referencing the *Solanum tuberosum* UniProt database (Tyanova et al., 2015). Trypsin specificity was set to allow up to three missed cleavages. Variable modifications included methionine oxidation and pyroglutamate formation, while carbamidomethylation of cysteine was set as a fixed modification.

Identification criteria included a minimum peptide length of five amino acids, at least one peptide (razor or unique), a precursor mass tolerance of 10 ppm, and a fragment ion mass tolerance of ± 0.2 Da. A false discovery rate (FDR) of 0.01% applied at both peptide and protein levels, with a maximum peptide posterior error probability set to 1. The derived peak list generated by Quant.exe (the first part of MaxQuant) was searched using the Andromeda search engine integrated into the MaxQuant against the *Solanum tuberosum (potato)* fasta file downloaded from the UNIPROT web site. MaxQuant.exe (the first module of MaxQuant) was searched with the Andromeda search engine, integrated into MaxQuant, against the *Solanum tuberosum* (potato) FASTA file obtained from the UniProt database. MaxQuant files were subsequently analyzed using Perseus software (version v2.1.5.0) (Tyanova et al., 2016). Within Perseus, proteins identified only by site, annotated as contaminants or listed as reverse were removed. The intensity values for each protein group were transformed using a log2(*x*) function. Protein entries with fewer than three valid values were excluded, and missing values were replaced by zero. To assess differences in the protein abundance between two conditions, a “two-sample” test with a permutation-based FDR and *q*-value estimation was performed. Proteins were considered differentially expressed when *t*-test adjusted *p*-value (FDR) was< 0.05. Finally, the STRING database (https://string-db.org/) was used to

integrate all known and predicted protein-protein associations, including both physical interactions and functional links, with a minimum required interaction score of 0.4. KEGG PATHWAY was a collection of manually drawn pathway maps representing our knowledge of the molecular interaction, reaction and relation networks (https://www.kegg.jp/blastkoala/).

## 3.4 Untargeted metabolomics

### 3.4.1 Chemicals

All reagents and standards used were mass spectrometry grade. Acetonitrile, water and methanol were obtained from Merck (Darmstadt, Germany). Formic acid was purchased from Sigma-Aldrich (St. Louis, MO). Authentic compounds for the identification of primary and secondary metabolites, were obtained from Sigma-Aldrich (St. Louis, MO, USA).

### 3.4.2 Liquid chromatography high resolution tandem mass spectrometry (LC-MS/MS)

Potato samples were freeze-dried and crushed to obtain a fine powder. Metabolites were extracted through a modified protocol by Troise et al. (2025). Briefly, 100 mg of pulverized freeze-dried samples were mixed with 3 mL of a methanol/water/formic acid 70/29,5/0.5 (v:v:v) solution in 5 mL microcentrifuge tubes. Hydroalcoholic suspensions were incubated in an ultrasonic bath for 15 min at 20°C then vortexed 5 min at 750 rpm. After centrifugation at 18000 xg, for 5 min, 4°C each supernatant was diluted 5 times in water and transferred to autosampler vial. Quality control samples were prepared by mixing all the experimental samples in equal amounts, while a procedural blank was also prepared in parallel with potato samples to ensure background correction in data processing and minimize solvent effects. Metabolites screening and phytochemicals analysis was achieved by reversed phase liquid chromatography high resolution tandem mass spectrometry (LC-MS/MS) employing an Exploris 120 quadrupole Orbitrap high-resolution mass spectrometer interfaced to a Vanquish Core liquid chromatographic system (Thermo Fisher Scientific, Bremen, Germany). The column was a biphenyl core shell column (Kinetex biphenyl, 100 x 2.1, 2.6 µm, Phenomenex, Torrance, CA) at a controlled temperature of 35°C. The elution solvents were 0.1% formic acid in water (solvent A) and acetonitrile (solvent B) with a 20 min elution gradient at flow rate of 0.3 mL/min: 0-0.5 min (5% B), 0.5-1.5 min (15% B), 1.5-10 min (40% B), 10-15 min (70% B), 15-16 min (95% B). Sample runs were acquired in positive and negative polarity switching mode, scanning the ions in full scan mode in the m/z range 120-1500. The conditions for HRMS were set as follows: heated electrospray interface (H-ESI) static spray voltage was -3.2 kV in negative ions and 3.5 kV for positive ion; ion transfer tube temperature, 300°C; vaporizer temperature, 320 °C; sheath gas flow, 50 arbitrary units; auxiliary gas flow, 10 arbitrary units. The analyser resolution was set at 60000 (FWHM at *m/z* 200) and data acquired in profile mode with a normalized AGC target of 100%. Compound identification was achieved through analysis of quality controls spiked with a mixture of analytical standards (Phytomols, phytochemical standard mix, Merck, Darmstadt, Germany) and scanning ions across differential ranges in both positive and negative data-dependent scan mode (ddMS2). To add a fragmentation spectrum to the isotopic patterns and molecular formulas, as the full scan acquisition described above (*m/z* 120-1500), four intertwined differential scan ranges (*m/z* 115-450, *m/z* 440-750, *m/z* 740-1050*, m/z* 1040-1510) at same resolution in the same chromatographic condition. For these experiments an isolation window of m/z 1.5 and a normalized collision energy at 20, 45 and 60% were used. Intensity threshold was fixed at 50000 (area counts), the dynamic exclusion was set by considering as time window 3.5 s and a mass tolerance of 5 ppm. A targeted mass exclusion list was obtained in the full scan mode by injecting blank procedural samples and blank solvents. EASY-IC, employing fluoranthene in both positive ion mode (*m/z* 202.0777 [M]^+^) and negative ion mode (*m/z* 202.0788 [M]^-^), was utilized to enhance mass centring and accuracy in Full Scan and ddMS2 mode (scan to scan acquisition). Profile data were analysed using Xcalibur 4.5 (Thermo Fisher Scientific, Waltham, MA), while fragmentation spectra were recorded by using Free Style software (v. 1.8, Thermo Fisher Scientific, Waltham, MA).

### 3.4.3 Untargeted metabolomics

All raw data files (samples, quality controls, procedural blank and identification runs) in Full Scan and ddMS2 were processed using Compound Discoverer 3.3 (Thermo Fisher Scientific, San José, CA). To evaluate differences between “LED” and “CTRL” samples, an untargeted metabolomic workflow based on the identification of primary and secondary metabolites was applied. This software was able to obtain the consequential retention time alignment, the background removal and unknown metabolite detection for the interrelated compounds grouping according to their chemical features. Compounds identification was based on, exact masses, formulas and fragmentation spectra against MS fragmentation pattern of metabolites available on databases including mzCloud, ChemSpider, phenol-explorer, natural compounds Atlas and KEGG. For some metabolites the putative identification was confirmed by analysis of standards listed in table M1. The software was also used to perform a differential analysis via Volcano Plot and a Principal Component Analysis (PCA). For volcano plots between “LED” an “CTRL” samples, p-values were adjusted by Benjamini-Hochberg algorithm.

# 4 Supplemental references

Chen, S., Zhou, Y., Chen, Y., and Gu, J. (2018). fastp: an ultra-fast all-in-one FASTQ preprocessor. *Bioinformatics* 34:i884–i890.

Dobin, A., Davis, C. A., Schlesinger, F., Drenkow, J., Zaleski, C., Jha, S., Batut, P., Chaisson, M., and Gingeras, T. R. (2013). STAR: ultrafast universal RNA-seq aligner. *Bioinformatics* 29:15–21.

Li, L., Luo, Z., Huang, X., Zhang, L., Zhao, P., Ma, H., Li, X., Ban, Z., and Liu, X. (2015). Label-free quantitative proteomics to investigate strawberry fruit proteome changes under controlled atmosphere and low temperature storage. *J. Proteomics* 120:44–57.

Liao, Y., Smyth, G. K., and Shi, W. (2014). featureCounts: an efficient general purpose program for assigning sequence reads to genomic features. *Bioinformatics* 30:923–930.

Rau, A., Gallopin, M., Celeux, G., and Jaffrézic, F. (2013). Data-based filtering for replicated high-throughput transcriptome sequencing experiments. *Bioinformatics* 29:2146–2152.

Robinson, M. D., McCarthy, D. J., and Smyth, G. K. (2010). edgeR : a Bioconductor package for differential expression analysis of digital gene expression data. *Bioinformatics* 26:139–140.

Troise, A. D., De Pascale, S., Piluzza, G., Re, G. A., Sanna, F., Scaloni, A., and Sulas, L. (2025). Metabolite variations in sulla shoots induced by drought stress. *J. Sci. Food Agric.* 105:1069–1079.

Tyanova, S., Temu, T., Carlson, A., Sinitcyn, P., Mann, M., and Cox, J. (2015). Visualization of LC‐MS/MS proteomics data in MaxQuant. *Proteomics* 15:1453–1456.

Tyanova, S., Temu, T., Sinitcyn, P., Carlson, A., Hein, M. Y., Geiger, T., Mann, M., and Cox, J. (2016). The Perseus computational platform for comprehensive analysis of (prote)omics data. *Nat. Methods* 13:731–740.

Wang, L., Wang, S., and Li, W. (2012). RSeQC: quality control of RNA-seq experiments. *Bioinformatics* 28:2184–2185.

Yu, G., Wang, L.-G., Han, Y., and He, Q.-Y. (2012). clusterProfiler: an R package for comparing biological themes among gene clusters. *Omi. A J. Integr. Biol.* 16:284–287.
